# Homopolish: a method for the removal of systematic errors in nanopore sequencing by homologous polishing

**DOI:** 10.1186/s13059-021-02282-6

**Published:** 2021-03-31

**Authors:** Yao-Ting Huang, Po-Yu Liu, Pei-Wen Shih

**Affiliations:** 1 Department of Computer Science and Information Engineering, National Chung Cheng University, Chiayi, Taiwan; 2 Department of Infectious Diseases, Taichung Veterans General Hospital, Taichung, Taiwan; 3 Rong Hsing Research Center for Translational Medicine, National Chung Hsing University, Taichung, Taiwan; 4 Ph.D. Program in Translational Medicine, National Chung Hsing University, Taichung, Taiwan

**Keywords:** Genome polishing, Nanopore sequencing

## Abstract

Nanopore sequencing has been widely used for the reconstruction of microbial genomes. Owing to higher error rates, errors on the genome are corrected via neural networks trained by Nanopore reads. However, the systematic errors usually remain uncorrected. This paper designs a model that is trained by homologous sequences for the correction of Nanopore systematic errors. The developed program, Homopolish, outperforms Medaka and HELEN in bacteria, viruses, fungi, and metagenomic datasets. When combined with Medaka/HELEN, the genome quality can exceed Q50 on R9.4 flow cells. We show that Nanopore-only sequencing can produce high-quality microbial genomes sufficient for downstream analysis.

**Supplementary Information:**

The online version contains supplementary material available at (10.1186/s13059-021-02282-6).

## Background

Third-generation long-read sequencing is an essential technology for the reconstruction of complete genomes in many species within the biosphere. Oxford Nanopore Technology (ONT) is one of the major providers in third-generation sequencing, which is being used for the telomere-to-telomere reconstruction of the human genome [1, 2]. Although the ultra-long reads of ONT have demonstrated their power in assembly contiguity through large and complex repeat regions, their assembly accuracy (∼ 85–92%) has been criticized when compared with Illumina or PacBio High-Fidelity (HiFi) sequencing (∼ 99%), owing to the omission of important protein-coding genes [3]. As a consequence, hybrid Illumina/Nanopore sequencing and assembly are required for producing a high-quality genome which possesses both satisfactory contiguity and accuracy [4].

The accuracy of Nanopore sequencing has improved year by year thanks to new basecalling algorithms (e.g., from Albacore, Guppy, to Bonito) and flow cells (e.g., from R9.4 to R10.3). For instance, the production basecaller (Guppy v3.6) has claimed a 1–2% increase in accuracy (∼ 97%) over its previous version. However, the genome quality of Nanopore-only sequencing is far from satisfactory owing to a substantial number of systematic errors. Consequently, all the long-read assemblers (e.g., Canu, miniasm, Flye, Shasta) require a polishing stage for further improving the genome quality [5–8]. At the beginning, signal-based polishing (e.g., Nanopolish) was adopted, whereas potential erroneous loci are re-basecalled from raw signals [9]. However, this method is time-consuming and disk-demanding for both the processing and storing of a huge amount of signals. Currently, read-based polishing has become mainstream as only reads rather than signals are required. Thanks to the advances in combinatorial and deep learning algorithms, these programs are not only faster, but their accuracy is also higher than signal-based approaches. Below, we focus on two state-of-the-art read-based polishing pipelines: Racon/Medaka and MarginPolish/HELEN.

Racon is one of the most popular polishing programs [10]. It first carefully selects high-quality parts of the reads, which are used for polishing the genome via partial order alignment (POA) with vectorization. Although many errors are corrected by Racon, a substantial amount of systematic errors are remaining in the genome because the correct allele is a minority at these loci (Additional file 1: Fig. S1). As a consequence, Oxford Nanopore Inc. developed Medaka, which is based upon a bidirectional long-short-term memory (LSTM) trained for erasing systematic errors uncorrected by Racon. To date, polishing first by Racon and subsequently followed up by Medaka is the officially recommended protocol (i.e., Pomoxis) for genomes assembled solely from Nanopore sequencing.

Recently, a new polishing pipeline named MarginPolish/HELEN has drawn attention by displaying competitive accuracy when compared with the Racon/Medaka pipeline [8]. MarginPolish uses a hidden Markov model to collect alignment statistics and then generates a weighted POA graph for consumption by HELEN. Subsequently, HELEN incorporates a multi-task recurrent neural network (RNN) that utilizes both the contextual genomic features and POA weights to predict with high accuracy a nucleotide base and run length for each genomic position.

However, these polishing protocols still fail to guarantee that a high-quality genome can be produced. In reality, only a ∼ Q30 (99.9%) consensus accuracy can be reliably obtained, implying that quite a few genes would be missed in downstream analysis [3, 11]. This unsatisfactory quality is partly due to species-specific DNA modifications. Theoretically, these errors could be avoided by training a basecaller specific for each species (e.g., Taiyaki) [11]. But in a practical sense, it is infeasible to train thousands of models for all the species in the biosphere. In metagenomic sequencing, training of a basecaller for a specific species is not an option. We observed that systematic errors are often uncorrected by existing polished tools, which are mainly indel errors in homopolymers and lead to reading frame shifts in protein-coding genes. These systematic errors, though difficult to be fixed solely from reads, can be easily corrected via the homologous sequences extracted from closely related genomes (Additional file 1: Fig. S2). Consequently, existing polishing models can be further improved according to the degree of conservation within the homologous regions.

This paper develops a novel polishing tool (named Homopolish), which is based upon a support vector machine (SVM) trained for distinguishing between sequencing errors and strain variations using homologous sequences. Due to its carefully engineered features, the results indicate that Homopolish outperforms the state-of-the-art Medaka and HELEN pipelines over a variety of microbial genomes, including metagenomic, bacterial, viral, and fungal genomes.

## Results

**Fig. 1:**
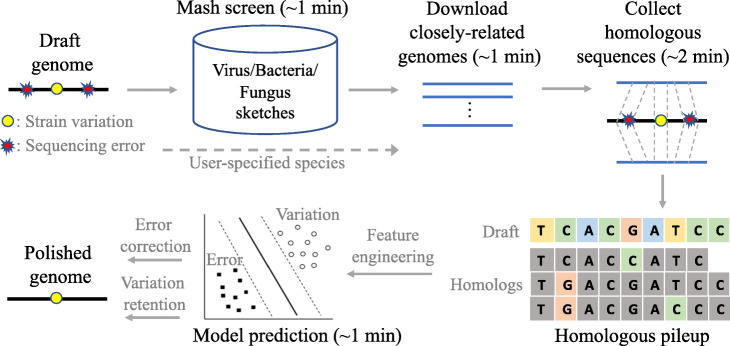
Illustration of Homopolish workflow. Homopolish retrieves closely related genomes by screening MinHash sketches of NCBI RefSeq microbial genomes. Homologs within these genomes were extracted by genome alignments. An SVM was trained for the removal of errors and retention of variations by mining features from the homologous pileup

Homopolish is based on an SVM trained for distinguishing Nanopore systematic errors (indels in particular) from interstrain variations according to the degree of conservation among homologs (see Fig. 1 and the “Methods” section). The homologs are extracted from closely related genomes and mapped onto the draft genome. A set of carefully engineered features are then generated from the homologous alignments for classification by the SVM (i.e., errors correction or variation retention).

Herein, we compare Homopolish with two state-of-the-art polishing pipelines (Racon/Medaka and MarginPolish/HELEN) [8, 10]. These programs are evaluated using public and in-house sequenced metagenomic/isolate datasets, including bacteria, virus, and fungi (Additional file 2: Tables S1-S3, see the “Methods” section). The Nanopore reads of all datasets are first assembled into draft genomes using Flye or MetaFlye (Additional file 2: Tables S4-S6) [7, 12]. These draft genomes are then first corrected by either Racon or MarginPolish for the removal of random sequencing errors. Subsequently, the remaining systematic errors are polished by Medaka, HELEN, and Homopolish. The accuracy of the polished genome is measured by (median) *Q* scores, number of mismatches, number of insertions, and number of deletions calculated via fastmer [13].

### Comparison of genome quality on R9.4 metagenomic datasets

**Fig. 2:**
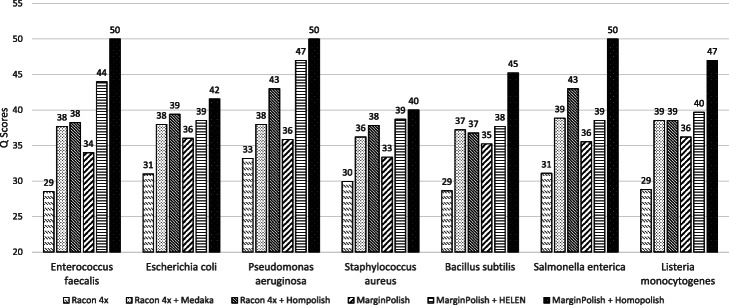
Comparison of genome quality on the R9.4 metagenomic dataset. Comparison of genome quality (*Q* score) polished by Racon, Medaka, Homopolish (R), MarginPolish, HELEN, and Homopolish (M) on the metagenomic dataset from Zymo Microbial Community Standard. Medaka and Homopolish (R) are run after Racon. HELEN and Homopolish (M) are invoked after MarginPolish. Homopolish (M) and Homopolish (R) achieve the highest accuracy in most bacteria

We first compare Medaka over Racon, Homopolish (R) over Racon, HELEN over MarginPolish, and Homopolish (M) over MarginPolish through the use of a metagenomic dataset (Zymo Microbial Community Standard) sequenced by R9.4 flow cells [14]. Figure 2 lists the *Q* scores of all programs regarding seven bacteria within the metagenomic sample, with the numbers of insertions, deletions, and mismatches being found in Additional file 2: Table S7. Homopolish (R) and Homopolish (M) (∼ Q38–Q50) outperform Medaka (∼ Q36–38) and HELEN (∼ Q37–Q46) in most bacteria. Homopolish (M) achieves Q50 (99.999%) in *Enterococcus faecalis*, *Pseudomonas aeruginosa*, and *Salmonella enterica*. The only exception is *Bacillus subtilis* (Q36.78 for Homopolish and Q37.21 for Medaka), which is due to mismatches uncorrected by Homopolish, although indels are greatly reduced (Additional file 1: Fig. S3). In general, systematic errors are greatly reduced by Medaka, HELEN, and Homopolish in comparison with those removed by Racon and MarginPolish. Moreover, the results based upon MarginPolish (i.e., HELEN and Homopolish (M)) are superior to those based upon Racon (i.e., Medaka and Homopolish (R)).

**Fig. 3:**
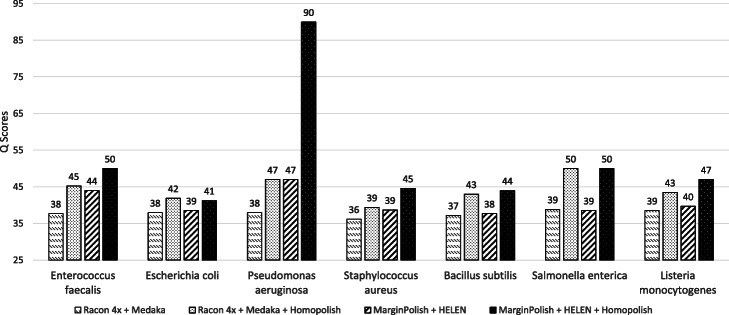
Genome quality of combining Homopolish with Medaka or with HELEN in the metagenomic dataset. Comparison of genome quality (*Q* score) polished by Medaka, Medaka+Homopolish, HELEN, and HELEN+Homopolish on the metagenomic dataset. Medaka and HELEN can be both further improved by Homopolish

As mismatch errors are not corrected by Homopolish, Homopolish could in principle achieve even higher accuracy when combined with Medaka or HELEN. Figure 3 plots the median *Q* scores of invoking Homopolish after Medaka and HELEN polishing. The accuracy of Medaka and HELEN is both further improved by Homopolish (Q40–Q90). For instance, Homopolish after Medaka now reaches Q50 on *S. enterica* and exceeds Q40 for most bacteria. In general, Homopolish with HELEN (Q41–Q90) outperforms that with Medaka (Q39–Q50). In particular, Homopolish after HELEN achieves Q90 on *P. aeruginosa* and Q50 on both *E. faecalis* and *S. enterica*.

Table 1 lists the numbers of mismatches, insertions, and deletions of the *P. aeruginosa* genome polished by all programs, whereas those of the other six bacteria can be found in Additional file 2: Table S7. The quality of the draft genome (assembled and polished by Flye) is only approximately Q26, and the errors are dominated by 14,394 insertions. Racon removed quite a few insertion errors at the cost of producing many false deletion errors (from 439 up to 2417). Medaka corrected most of the deletion errors produced by Racon (from 2417 down to 324), as well as a few mismatches (from 622 to 476). Homopolish significantly erased most insertion and deletion errors (from 538 and 2417 down to 24 and 128, respectively) left by Racon. These phenomena are largely the same when Homopolish is compared with HELEN over MarginPolish. HELEN is superior at correcting mismatch errors and outperforms Medaka in all metrics. Again, Homopolish cleans the majority of indel errors left by MarginPolish. When Homopolish is combined with either Medaka or HELEN, the genome quality can be further elevated to Q50 and Q90, respectively, because both mismatch and indel errors are significantly removed by each program. The advantage of combining Homopolish with either Medaka or HELEN can also be seen in other bacteria (Additional file 2: Table S7).

**Table 1:** *Q* scores and numbers of mismatch, insertion, and deletion errors of *P. aeruginosa* in the metagenomic dataset

Methods	Avg. *Q* score	Median *Q* score	Mismatches	Insertions	Deletions
Flye	26.39	26.5	738	14394	439
Racon 4x	32.78	33.19	622	538	2417
Racon 4x + Medaka	36.54	37.96	**476**	704	324
Racon 4x + Hompolish	**39.43**	**43.01**	622	**24**	**128**
MarginPolish	34.93	35.85	449	1096	637
MarginPolish + HELEN	**41.48**	46.99	**149**	78	256
MarginPolish + Homopolish	40.74	**50**	440	**7**	**126**
Racon 4x + Medaka + Homopolish	40.55	46.99	474	**4**	**120**
MarginPolish + HELEN + Homopolish	**42.81**	**90**	**205**	5	146

### Comparison of polishing accuracy on bacterial isolates

**Fig. 4:**
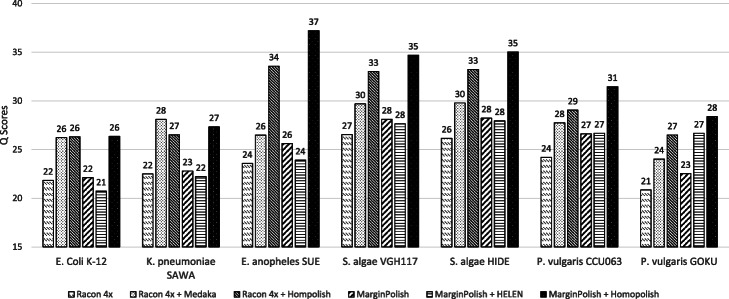
Comparison of genome quality on the R9.4 isolate dataset. Comparison of genome quality (*Q* score) polished by Racon, Medaka, Homopolish (R), MarginPolish, HELEN, and Homopolish (M) on seven bacterial isolates. Medaka and Homopolish (R) are run after Racon. HELEN and Homopolish (M) are invoked after MarginPolish

Next, we compared these programs over a set of bacterial isolates sequenced at an earlier stage (see the “Methods” section). These data were mainly sequenced prior to 2018 and basecalled by Albacore and/or early Guppy basecaller, which exhibit a lower quality when compared with the previous metagenomic dataset (called by Guppy 2.2). Theoretically, old sequencing data can be re-basecalled using new algorithms (e.g., Guppy 4.0 or Bonito). But for practical purposes, particularly for labs outsourcing the sequencing and assembly, this is very troublesome as re-basecalling is computationally demanding without GPU. We show that Homopolish can improve the quality of Nanopore-only genomes produced by an old basecaller (e.g., Albacore) without the need of re-basecalling. Figure 4 plots the *Q* scores of all programs across seven isolates. Similarly, Homopolish (Q26–Q33) outperforms Medaka with Racon (Q23–Q29) in nearly all datasets except for *Klebsiella pneumoniae* in which mismatches (not corrected by Homopolish) are the major error source (Additional file 1: Fig. S4). In comparison with HELEN over MarginPolish (Q20–Q27), Homopolish (M) also demonstrates superior accuracy (Q26–Q37) across all isolates. Unexpectedly, the accuracy of HELEN is not only lower than Medaka but also lower than its preprocessor MarginPolish in most datasets (i.e., *Escherichia coli*, *K. pneumoniae*, *Elizabethkingia anophelis*, and *Shewanella algae*), which conflicts with the previous metagenomic results. We hypothesize that HELEN may overfit the previous metagenomic dataset since the majority of its training data come from the same source (ZymoBIOMICS) [14].

**Fig. 5:**
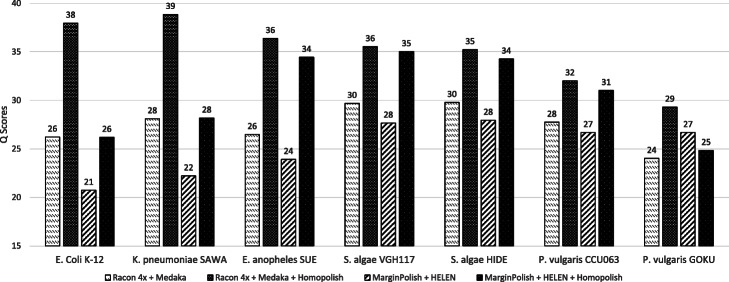
Genome quality of combining Homopolish with Medaka or with HELEN in the isolate dataset. Comparison of genome quality (*Q* score) polished by Medaka, Medaka+Homopolish, HELEN, and HELEN+Homopolish on the isolate dataset. Although Homopolish can improve Medaka or HELEN, the degree of improvement is decreased from common (e.g., *E. coli*) to rare species (e.g., *P. vulgaris*)

Similarly, we have evaluated whether Homopolish can achieve even higher accuracy when combined with either Medaka or HELEN over these isolates. Figure 5 illustrates the *Q* scores of Homopolish run after Medaka or HELEN correction. When combined with Medaka, Homopolish obtains a much higher accuracy (Q29–Q38), significantly better than those of original Medaka (Q23–Q29). Although Homopolish with HELEN also improves accuracy from Q20–Q27 to Q24–Q35, it is inferior to that with Medaka. Table 2 lists the *Q* scores, number of mismatches, insertions, and deletions polished by each program of *E. anophelisSUE*, while those of other isolates can be found in Additional file 2: Table S8. The initial genome quality of Flye is low, containing 18,718 insertions and 5039 deletions. Although Racon, Medaka, MarginPolish, and HELEN reduce the insertion errors, the number of deletion errors all increase (i.e., from 5039 to 7891–11,753), implying quite a few false-positive corrections. On the contrary, only Homopolish does not introduce more false deletions into the draft genome (i.e., from 5039 to 435–443). In particular, HELEN performs the worst due to increasing indel errors from its preprocessor MarginPolish in most isolates (Additional file 2: Table S8). Overall, only Homopolish can reduce most indel errors without much side effects. When combined with Medaka for removing both mismatch and indel errors, Homopolish achieves an expected higher accuracy (from Q33 to Q36). However, the combination with HELEN (Q34) is not better than running Homopolish directly on top of MarginPolish (Q37).

**Table 2:** *Q* scores and numbers of mismatch, insertion, and deletion errors of *E. anophelis* *SUE* in the isolate dataset

Methods	Avg. *Q* score	Median *Q* score	Mismatches	Insertions	Deletions
Flye	22.25	22.2	1363	18718	5039
Racon 4x	23.57	23.57	1163	6097	11215
Racon 4x + Medaka	26.43	26.48	**566**	1114	7891
Racon 4x + Hompolish	**33.11**	**33.57**	1224	**384**	**443**
MarginPolish	25.57	25.61	**389**	1055	10198
MarginPolish + HELEN	23.89	23.91	669	4614	11753
MarginPolish + Homopolish	**35.8**	**37.21**	447	**222**	**435**
Racon 4x + Medaka + Homopolish	**35.32**	**36.38**	**611**	**216**	**407**
MarginPolish + HELEN + Homopolish	33.57	34.44	723	342	774

### Comparison of correction accuracy on viral and fungal genomes

To demonstrate the capability of Homopolish on polishing other microbial genomes, these programs were further tested on one viral genome (*Lambda phage*) and one fungal genome (*S. cerevisiae*). As shown in Table 3, the quality of the draft viral genome is approximately Q24. Racon with Medaka improved the quality to Q36. Homopolish achieved the highest accuracy when combined with either Racon or Medaka (∼ Q38–39). Again, the accuracy of HELEN is not only the worst, but it is also lower than the original draft genome (from ∼ Q24 to ∼ Q20), owing to new mismatches and deletion errors falsely corrected. As for the fungal genome (*S. cerevisiae*), the quality of the initial genome was approximately Q20. Correction via Racon with Medaka improved the quality to Q23. The highest accuracy is once more achieved by Homopolish when combined with either Medaka or MarginPolish (∼ Q32). Similarly, HELEN obtained the lowest accuracy in comparison with the others. These results indicate that Racon, Medaka, and Homopolish are robust to polished viral, bacterial, and fungal genomes while HELEN is less reliable when compared with the others.

**Table 3:** *Q* scores and numbers of mismatch, insertion, and deletion errors on *Lambda* *phage* and *Saccharomyces* *cerevisiae*

Species	Methods	Avg. *Q* score	Median *Q* score	Mismatches	Insertions	Deletions
*Lambda phage*	Flye	24.17	24.17	7	177	2
	Racon 4x + Medaka	36.44	36.44	**4**	5	2
	Racon 4x + Hompolish	**39.07**	**39.07**	**4**	**1**	**1**
	Racon 4x + Medaka + Homopolish	**38.4**	**38.4**	**4**	2	**1**
	MarginPolish + HELEN	19.95	19.95	123	16	144
	MarginPolish + Homopolish	37.31	37.31	6	2	**1**
	MarginPolish + HELEN + Homopolish	21.99	21.99	169	10	18
*S. cerevisiae*	Flye	20.43	20.52	2715	75290	8459
	Racon 4x + Medaka	22.89	23.55	2345	44979	3232
	Racon 4x + Hompolish	27.84	30.64	4683	10556	1042
	Racon 4x + Medaka + Homopolish	**28.72**	**32.58**	2374	9917	**928**
	MarginPolish + HELEN	20.38	20.71	3123	45701	26865
	MarginPolish + Homopolish	**28.7**	**32.68**	**2007**	**9547**	1817
	MarginPolish + HELEN + Homopolish	24.86	26.09	3449	8997	15572

### Comparison of genome quality on R10.3 flow cells

**Fig. 6:**
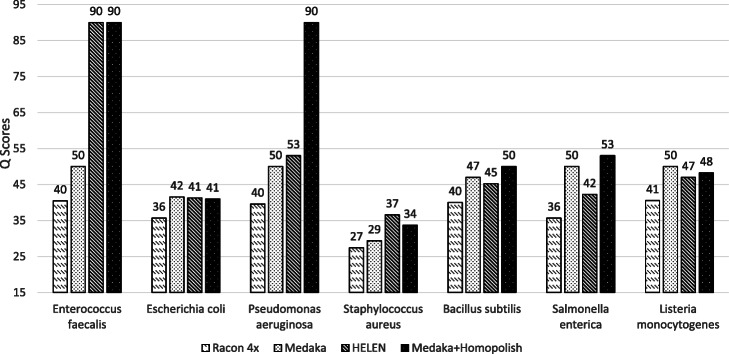
Comparison of genome quality on the R10.3 metagenomic dataset Comparison of genome quality (Q score) polished by Racon, Medaka, HELEN, and Homopolish on the R10.3 metagenomic dataset from Zymo Microbial Community Standard. HELEN was run after MarginPolish. Medaka was run after Racon, and Homopolish was invoked after Medaka

Finally, we evaluated Racon, Medaka, HELEN, and Homopolish on one public metagenomic dataset (Zymo Microbial Community Standard). The accuracy of the R10.3 flow cell is higher than the R9.4 version, albeit at the cost of reduced throughput. Figure 6 plots the *Q* scores of Racon, Medaka, HELEN, and Homopolish for the seven bacteria in the R10.3 metagenomic dataset. Additional file 2: Table S9 lists the numbers of mismatches, insertions, and deletions of each program as applied to these bacteria. The quality of draft genomes ranged from Q28 to Q42, which is indeed better than that of R9.4 (Q22–Q26) (Additional file 2: Table S7). Unexpectedly, the genomes polished by Racon were not improved (Q27–Q40) when compared with the draft genome assembled by Flye. The Medaka-polished genomes exhibited significantly higher quality (Q30–Q50). HELEN obtained the highest quality of two strains (i.e., Q90 on *E. faecalis* and Q36 on *S. aureus*) when compared with others. Homopolish further improved the Medaka-polished genomes to Q33–Q90 and obtained the highest quality of four strains (e.g., Q90 on *E. faecalis* and *P. aeruginosa*). When testing Homopolish and Medaka over the public (*E. coli* K12 MG1655 sequenced by R10.3 flow cell (Additional file 2: Table S9), Homopolish also improved Medaka from Q46 to Q90. Consequently, these results suggest that Homopolish can also improve the genome quality of Medaka on R10.3 flow cells.

## Discussion

### Limitations of homologous polishing

Homopolish relies on homologous sequences retrieved from closely related genomes for the correction of Nanopore systematic errors. Thus, the efficiency is related to the abundance of related genomes in NCBI (i.e., common or rare). When polishing rare species (e.g., *Proteus vulgaris* CCU063 in Fig. 5), although Homopolish still improved the quality of these two genomes (e.g., Q27 to Q32), the degree of improvement is lower than polishing common species (e.g., Q26 to 38 in *K. pneumoniae*). In the bacterial isolate dataset, the genome quality declines from common (e.g., Q38 in *E. coli*), less common (e.g., Q35 in *S. algae*), to rare species (e.g., Q29 in *P. vulgaris*).

Second, the trained model aims to remove errors within conserved regions and retain strain variations free of selection pressure. As a result, the errors within non-coding regions may tend to be uncorrected. Table 4 compares the numbers of errors in coding and non-coding regions before and after running Homopolish. Although the correction ratios in the coding regions are higher as expected (70–95%), the errors in the non-coding regions are reduced at various degrees (28–97%). The large variance of correction ratios in non-coding regions is possibly due to species-specific selection pressures. Therefore, the efficacy of Homopolish in non-coding regions will depend on the underlying selection pressure of each species. We note that Homopolish has not been tested on non-compact genomes, in which non-coding regions represent an important part of the genome. These genomes, in fact a large proportion of eukaryotic genomes, could not be corrected to the same level as the small and compact microbial genomes.

**Table 4:** Comparison of the numbers of errors in coding and non-coding regions before and after running Homopolish using the Zymo R9.4 dataset

	Coding regions errors			Non-coding regions errors		
	Before	After	Correction ratio	Before	After	Correction ratio
*B. subtilis*	446	80	82.06%	232	165	28.88%
*E. faecalis*	244	21	91.39%	176	34	80.68%
*S. aureus*	306	38	87.58%	100	17	83.00%
*L. monocytogenes*	160	19	88.13%	80	26	67.50%
*P. aeruginosa*	666	30	95.50%	279	8	97.13%
*S. enterica*	469	66	85.93%	118	13	88.98%
*E. coli*	544	163	70.04%	201	141	29.85%

DFAST was used for the annotation of coding/non-coding regions in each genome

Third, the correction efficacy of Homopolish may be reduced on plasmids shaped by mobile genetic elements (e.g., integrons), which exhibit little or no structural conservation across plasmids. As a consequence, the homologs can not be obtained because no closely related plasmids can be found by Mash. Moreover, the MinHash sketches used for the identification of closely related genomes are compiled from nearly complete genomes. When polishing highly fragmented assembly (e.g., due to low sequencing coverage), the species identification stage (i.e., Mash screen) becomes less reliable. Consequently, the user should specify the genus or species name when using Homopolish for correcting highly fragmented assembly.

### Preservation of strain variations

Because the correction material of Homopolish is homologs instead of reads, the genome variability (e.g., phylogenetic distance) between the polished and related genomes might decrease. To prove that the strain variations are well preserved, we compare the whole-genome phylogenies before and after Homopolish correction using common and rare species. The twenty most closely related genomes (> 99% Mash identity) of two common species (*P. aeruginosa* and *S. enterica* in the Zymo dataset) were retrieved for whole-genome phylogeny reconstruction (see the “Methods” section). Although their genetic distance is very close, the phylogenies remain the same before and after Homopolish (Additional file 1: Figs. S15 and S16). Similarly, the phylogenies of two rare species (*P. vulgaris* CCU063 and GOKU) and their closely related genomes are also unchanged after Homopolish (Additional file 1: Figs. S12 and S13). Therefore, the phylogenetic distance between the polished and related genomes is well preserved.

Furthermore, most bacterial genomes contain pseudogenes (e.g., genes inactivated by true indels), which should not be corrected by homologs. We compared the pseudogenes (annotated by DFAST [15]) in the genomes polished by Medaka, Homopolish, and the truth genome (Additional file 1: Figs. S17 and S18). The Racon-Medaka pipeline produces excessive amounts of pseudogenes (e.g., 151–5298 in R9.4) due to quite a few uncorrected errors. On the contrary, the genomes polished by Homopolish yield not only much fewer pseudogenes (e.g., 10–126 in R9.4) but the numbers are also quite close to those of truth genomes (e.g., 12–136 in R9.4). Further investigation reveals that the minor discrepancies between Homopolished genomes and the truth genome (e.g., 62 vs 69 in *Bacillus*) are due to pseudogenes that have been missed before running Homopolish (e.g., misassembly). Consequently, most pseudogenes are successfully preserved by our method.

### Reference-free versus reference-based quality assessments

The assessment of genome quality is affected not only by uncorrected errors but also by misassemblies and structural variations (e.g., IS movement), which are too large to be corrected by existing polishing programs. In the isolate dataset, the references reconstructed via hybrid Illumina/Nanopore assembly are not the true underlying genomes, which also reduces the accuracy of reference-based assessment (e.g., *Q* scores by fastmer). Hence, we compared the (CheckM) genome completeness of Medaka and Homopolish, which is a widely used reference-free assessment [16] (Additional file 1: Fig. S10). The completeness of Homopolish (∼ 97–100%) is not only higher than that of Medaka (∼ 81–95%) but also quite close to that of truth genomes (∼ 97–100%). The same phenomenon can be also seen in the metagenomic dataset (Additional file 1: Fig. S11), implying the improvement of Homopolish is consistent in both reference-free and reference-based assessment.

Nevertheless, although reference-free assessments (e.g., CheckM/BUSCO/ideel) are less affected by misassemblies/structure variations, they are not sensitive enough to reflect the true accuracy of each program. We have observed that many genomes can obtain nearly 100% (CheckM) completeness even with only ∼ Q30 accuracy. On the other hand, reference-based assessments (e.g., fastmer), albeit affected by misassemblies/variations, can measure the accuracy at a higher resolution. We found that the median *Q* score is a relatively fair assessment of genome quality, which provides both sufficient resolution and robustness to misassemblies/structural variations.

### Future directions

While state-of-the-art polishing models are trained from Nanopore reads or signals (e.g., Nanopolish, Medaka, HELEN), we have shown that homologs conserved in closely related genomes provide valuable features for the detection of Nanopore systematic errors (e.g., frameshift being extremely rare within coding regions). Additionally, because Nanopore signals may be disturbed by species-specific DNA modifications, the construction of a universal model for polishing all species remains challenging. This work suggests that having a model aware of species-specific errors is possible, as long as both reads and homologs are incorporated into the underlying training framework (e.g., RNN in Medaka).

In terms of speed, because existing polishing algorithms (i.e., Medaka and HELEN) rely on the deep neural network, GPU acceleration is often required. Alternatively, a CPU is sufficient for Homopolish, as it is based upon an SVM (e.g., ∼ 5 min for polishing a bacterial genome). Although the neural network is theoretically suitable for learning non-trivial features, a set of manually inspected features in our model may be used by other developers for distinguishing Nanopore systematic errors.

The results indicated that the accuracy of R10.3 is indeed better than R9.4 on the same metagenomic dataset (Additional file 2: Tables S7 vs S9). Nevertheless, due to the use of an old version of Guppy (v2.2) basecaller in the R9.4 dataset, the read accuracy was still low (∼ Q9–Q10). However, recently, the accuracy of Nanopore reads has been significantly improved (e.g., ∼ Q13–14 by Guppy v3.6 over R9.4 flow cells), which should further enhance the genome quality as well (results not shown). In terms of the final genome quality, the accuracy gap between R9.4 and R10.3 is rather small (i.e., both can exceed Q50). As the throughput of R10.3 is currently lower than that of R9.4, R9.4 may still be preferred in sequencing projects requiring a large sequencing yield.

## Conclusion

This paper developed an SVM-based polishing model (named Homopolish) for the correction of Nanopore systematic errors using homologous sequences. In comparison with the state-of-the-art Medaka and HELEN, Homopolish displayed superior accuracy in bacteria, fungus, and virus on R9.4/R10.3 flow cells. The reported genome quality is expected to be further improved when using the latest basecaller (Guppy 4.0 or Bonito). Homopolish is currently only capable of polishing microbial genomes but not the majority of eukaryotic genomes, which require retraining the model and revising the methodology. In conclusion, we have proved that high-quality microbial genomes can be obtained through Nanopore-only sequencing using simply R9.4 flow cells, thus eliminating the need for Illumina hybrid sequencing.

## Methods

Homopolish corrects Nanopore systematic errors by an SVM trained for distinguishing sequencing errors from strain variations using homologous sequences. The genome to be polished is first screened against the virus, bacteria, or fungus genomes compressed in MinHash sketches, which are precompiled from NCBI RefSeq database using Mash [17]. Subsequently, closely related genomes are retrieved and aligned against the draft genome to extract conserved homologous sequences. Several features (e.g., homologous allele counts, homopolymer lengths, and homologous similarity) were extracted from the homologous alignments. Finally, an SVM is trained for distinguishing Nanopore systematic errors from interstrain variations. Only the systematic errors will be corrected according to the predicted base, while strain variations will be retained.

### Retrieval of homologous sequences via MinHash sketches

Given a genome *G* to be polished, Homopolish first identifies, downloads, and extracts homologous sequences from closely related genomes. In practice, whole-genome alignment against the entire NCBI RefSeq genome database is time-consuming and infeasible. Instead, these genomes are compressed into a reduced representation called Mash sketches [17]. Specifically, the microbial genomes in the NCBI RefSeq are downloaded and compiled into 1000 sketches per genome. The similarity of *G* against all RefSeq genomes can be thus estimated by Mash in seconds. Only the genomes with identity at least *p* (default 95%) are considered. Next, top *t* (default 20) genomic sequences are automatically downloaded from NCBI, which takes around 1 min for retrieving 20 bacterial genomes. Alternatively, Homopolish allows the user to specify the genus and/or species name of genome *G*, and *t* random genomes of the same genus and species will be downloaded from NCBI. To obtain the homologous sequences, these closely related genomes will be aligned against the genome *G* via minimap2 (with options asm5) [18], The alignment of these homologs against *G* (i.e., homologous pileup) will be further analyzed.

The genome quality (*Q* scores) with respect to parameter *t* (top *t* similar genomes) has been tested using fourteen bacteria (Additional file 1: Fig. S14). Although this parameter can be optimized for particular species (e.g., *t*=5 is highest in *Pseudomonas*), we did not see much difference after fine tuning. When *t*>20, the quality of most species deteriorates. Therefore, the default value (*t*=20) was chosen as it balances well for both common or rare species.

### Feature engineering and model training

A total of twelve features are extracted from the homologous alignment profile for distinguishing Nanopore systematic errors from interstrain variations. An SVM is trained for classifying each locus in the draft genome into errors or variations according to the twelve features. Only errors will be corrected while variations will be retained.

#### Feature engineering

At each locus of genome *G*, twelve features are extracted from the homologous alignment profile for distinguishing Nanopore systematic errors from interstrain variations. Note that we do not polish mismatch errors as they frequently occur in different strains of the same species. Besides, mismatches are less destructive within coding regions in comparison with indel errors. The first nine features are matched allele counts of A, T, C, G; inserted allele counts of A, T, C, G (Additional file 1: Fig. S5(a)); and deletion allele counts in homologous sequences (Additional file 1: Fig. S5(b)). These nine features aim to reflect the degree of conservation measured by allele frequencies within homologs (e.g., a single allele with 100% frequency indicates a very conservative locus). The 10th feature is termed homologous coverage (similar to read coverage), which reflects the confidence of homologous allele counts (i.e., higher coverage stands for higher confidence). The above ten features are min-max normalized into [0,1] intervals.

The eleventh feature encodes homopolymer length flanking the base being predicted. Additional file 1: Fig. S6(a) illustrates an insertion error at the start of consecutive five adenine bases (i.e., length five homopolymer). This feature is important as most Nanopore systematic errors are indels within homopolymers. Unexpectedly, we observed one-hot encoding of this feature achieves superior accuracy than naive min-max normalization (see Additional file 1: Fig. S7). We hypothesize this might owing to the side effects of basecalling algorithms (e.g., Guppy) overfitting particular lengths of homopolymers [19]. Hence, the length of homopolymer may be better encoded as a categorical instead of a numerical feature. To limit this feature with fixed dimensions, the homopolymer length is a skewed distribution (Additional file 1: Fig. S6(b)), whereas the majorities are lengths shorter than three and very few exceed ten. Consequently, the homopolymer length is one-hot encoded into ten categories {1, 2, 3, …, 8, 9, ≥10}.

The last feature aims to estimate the degree of conservation via homologous sequence similarity, which is manually inspected by Integrated Genomics Viewer [20]. The minor (instead of the major) allele among the homologous sequences is sometimes the true allele, which violates the assumption of the first ten allele count features, which will lead to false corrections. Further investigation indicated that the homologous sequences flanking these minor alleles are more similar to the ground-truth genome. For example (see Additional file 1: Fig. S8(a)), homologous sequence 1, though carrying minor allele, is perfectly identical to the ground-truth genome, while the other sequences are relatively dissimilar. This feature is also implemented by SNP calling algorithms (e.g., haplotype counts in freebayes and CNN in DeepVariant [21, 22].

In reality, because the ground-truth genome is not known in advance, the similarity of flanking sequences can only be estimated by aligning onto the draft genome *G*. Unfortunately, we observe the sequence similarity measured by *G* is sometimes indistinguishable between the major/minor alleles. For instance (in Additional file 1: Fig. S8(b)), the homologous sequences flanking the major (e.g., 2 and 3) and minor (e.g., 1 and 4) alleles both differ with the draft genome *G* by one insertion. Thus, their identities against the draft genome are completely the same. Interestingly, these ambiguous loci are mainly found in pairs and proximity (e.g., two insertion loci in Additional file 1: Fig. 8(a)(b)), and their major/minor alleles are mutually exclusive. Although we cannot explain this phenomenon, the minor (yet more similar with truth genome) alleles are usually the correct base, whereas the major alleles are not. As a result, the major and minor alleles at these paired loci are encoded as one and two, respectively, whereas other unpaired loci are encoded as zero. The SVM will be trained and learned to preserve the minor allele due to interstrain variations.

#### Model training

We aim to train an SVM able to distinguish between sequencing errors and strain variations. Instead of solving a binary classification problem, we found the accuracy is better by dividing errors and variations into seven classes: insertion of A, insertion of T, insertion of C, insertion of G, deletion, no insertion, and no deletion. The first five classes indicate errors to be corrected while the last two suggest variations to be retained. Because the deletion and insertion classes carry strong features in different dimensions (Additional file 1: Fig. S9), the accuracy can be improved by separating these distant classes in the feature space.

The class frequencies are quite imbalanced across the seven classes, whereas the no deletion class dominates all the others (≈ 30 million) (Additional file 2: Table S10). We remove duplicated feature vectors belonging to the no deletion class. The level of imbalance is greatly reduced although the samples of no deletion class are still the majority (∼ 38 thousand). The training data of the R9.4 model (basecalled by Guppy 2.2) include the following seven strains: *P. aeruginosa*, *E. coli*, *S. aureus*, *E. faecalis*, *K. pneumoniae*, *P. vulgaris VGH117*, and *P. vulgaris GOKU*, whereas the other seven bacterial strains, one fungus, and one virus are validation datasets not used during development. The training data of the R10.3 model includes four species: *P. aeruginosa*, *E. coli*, *S. aureus*, and *E. faecalis*, whereas the remaining four species are validation datasets. A total of 66,036 non-redundant samples in the training data were further split into a training and a test sets in a nine-to-one ratio. Subsequently, an SVM with the radial basis function (RBF) kernel (*c* = 1.0) is trained and evaluated in the test set. The macro average of precision, recall, and F1-score, as well as confusion matrix, of the test set can be found in Additional file 1: Fig. S20. During prediction, the genome is chopped into 10-kbp segments, which can be polished in a multithreaded environment in parallel.

### Collection and assembly of public datasets

The majority of datasets used in this study were retrieved from the public database. Two public metagenomic datasets (R9.4 and R10.3) of ZymoBIOMICS Microbial Community Standard were downloaded from Loman Lab [14] (Additional file 2: Table S3). Each Zymo metagenomic dataset includes eight bacteria and two yeasts. *Lactobacillus* was excluded because of unusually low quality (∼ Q20) possibly due to the wrong reference. The two yeasts were removed owing to highly fragmented assembly caused by low sequencing coverage. The reference genomes were obtained according to the ZymoBIOMICS instruction manual (Catalog No.D6300).

The Nanopore reads and reference genome of *E. coli* K12 sequenced by R10.3 were downloaded from [23]. The Nanopore reads of lambda phage virus were downloaded from NCBI Short Read Archive (SRA) (SRR12602365), whereas the reference genome (NC_001416.1) was obtained from RefSeq. The Nanopore reads of *S. cerevisiae* CEN.PK113-7D were downloaded from the SRA (SRR5989372), and the reference genome (GCA_000269885.1) was retrieved from RefSeq. The Nanopore reads of the public bacteria, virus, and fungus were first trimmed by PoreChop and assembled by Flye (v2.7) [7]. The metagenomic dataset was assembled by MetaFlye (v2.7) [12] (Additional file 2: Table S5).

### Hybrid assembly of six common and rare species

To compare the correction power on common and rare species, six additional bacterial isolates, *P. vulgaris* CCU063, *P. vulgaris* GOKU, *K. pneumoniae* SAWA, *E. anophelis* SUE, *S. algae* HIDE, and *S. algae* VGH117, were sequenced using both Illumina (MiSeq) and Nanopore (GridIon) platforms with ∼ 100-300 coverage (Additional file 2: Tables S1 and S2). The ground-truth genomes of the six bacteria were constructed by a hybrid assembly of Nanopore and Illumina reads via Unicylcer [4] (Additional file 2: Tables S4). The Nanopore-only genomes of the six isolates were assembled by Flye (v2.7) [7](Additional file 2: Table S6), whereas plasmids are excluded.

### Polishing workflow of Nanopore-only genomes

All the genomes assembled solely by Nanopore reads were first polished by either four rounds of Racon or MarginPolish for removal of random sequencing errors. The remaining systematic errors were then removed by either Medaka (v1.0.1), HELEN, or Homopolish. The quality of polishing genome (*Q* score), as well as numbers of insertions, deletions, and mismatches, were calculated by fastmer [13]. The genome completeness of polished genomes was computed by CheckM [16].

### Analysis of phylogeny, pseudogenes, and coding region bias

The protein-coding genes and pseudogenes in each genome were annotated by DFAST [15]. The numbers of sequencing errors within coding and non-coding regions were computed by bedtools (v2.2.28). For comparison of the whole-genome phylogenies of two common (*K. pneumoniae* and *S. enterica*) and two rare (*P. vulgaris*) species before and after running Homopolish, we retrieved 20 most closely related genomes with Mash identity >95*%*. Whole-genome phylogenies of each common or rare and its closely related genomes were reconstructed by REALPHY [24]. The phylogenetic tree was visualized via Interactive Tree Of Life (iTOL) [25].

## Supplementary Information

**Additional file 1** Additional file 1 includes Supplementary Figs. S1-20. Fig. S1. Illustration of Nanopore systematic errors. Fig. S2. Illustration of homologs for polishing systematic errors. Fig. S3. Numbers of indels and mismatches of *Bacillus*. Fig. S4. Numbers of indels and mismatches of *K pneumonia*. Fig. S5. Examples of feature vectors at insertion and deletion loci. Fig. S6. Illustration of systematic errors in homopolymers. Fig. S7. Comparison of min-max normalization with one-hot encoding. Fig. S8. Illustration of minor strain variations at multiple loci. Fig. S9. Comparison of feature vectors at deletion and insertion loci. Fig. S10. Comparison of genome completeness of seven bacteria. Fig. S11. Comparison of genome completeness of the R9.4 Zymo dataset. Fig. S12. Whole-genome comparison of the *P vulgaris* CCU063. Fig. S13. Whole-genome comparison of the *P vulgaris* GOKU. Fig. S14. Correlation of genome quality with respect to the number of related genomes. Fig. S15. Whole-genome comparison of the *P aeruginosa*. Fig. S16. Whole-genome comparison of the *S enterica*. Fig. S17. Comparison of pseudogenes in Zymo R9.4 dataset. Fig. S18. Comparison of pseudogenes in Zymo R10.3 dataset. Fig. S19. Comparison of PEPPER in Zymo R10.3 dataset. Fig. S20. Evaluation of model accuracy.

**Additional file 2** Additional file 2 includes Supplementary Tables S1-10. Table S1. Nanopore sequencing of six bacteria. Table S2. Illumina sequencing of six bacteria. Table S3. Nanopore sequencing of metagenomic datasets. Table S4. Hybrid assembly of six bacteria. Table S5. Metagenomic assembly of Zymo datasets. Table S6. Nanopore-only assembly of all isolates. Table S7. Comparison of polishing tools over the R9.4 Zymo dataset. Table S8. Comparison of polishing tools over the R9.4 bacterial dataset. Table S9. Comparison of polishing tools over the R10.3 Zymo dataset. Table S10. Comparison of label frequencies.

**Additional file 3** Additional file 3 includes the review history.

## Data Availability

Homopolish is freely available at https://github.com/ythuang0522/homopolish [26]. A cloud version (Code Ocean) can be accessed from https://codeocean.com/capsule/1612663/tree [27]. The version of source code used in the manuscript is deposited with DOI: 10.5281/zenodo.4301655 at https://zenodo.org/record/4301655#.X8dm9y8RpQI [28]. The hybrid Illumina/Nanopore assembled genomes of *K. pneumoniae* SAWA, *E. anophelis* SUE, *S. algae* VGH117, *S. algae* HIDE, *P. vulgaris* CCU063, and *P. vulgaris* GOKU were deposited at DDBJ/ENA/GenBank under the accession PKLG00000000, CP034247, CP032664, CP034246, CP032663, and CP034105, respectively [29–34]. Two public R9.4/10.3 metagenomic datasets (ZymoBIOMICS Microbial Community Standard) were downloaded from the Loman Lab [14]. The Nanopore reads of *E. coli* K12 sequenced by R10.3 were downloaded from the Albertsen Lab [23]. The Nanopore reads of lambda phage virus and *S. cerevisiae* CEN.PK113-7D were downloaded from NCBI Short Read Archive with accession numbers SRR12602365 and SRR5989372, respectively. The above data are also available at https://github.com/ythuang0522/homopolish/tree/master/data[35].
