# Supplementary material for: Homopolish: a method for the removal of systematic errors in nanopore sequencing by homologous polishing
Source: Genome Biol. 2021 Mar 31;22:95. doi: 10.1186/s13059-021-02282-6 (PMC8011154; doi:10.1186/s13059-021-02282-6)
Supplement: Supplementary file 1 — Additional file 1 Additional file 1 includes Supplementary Figs. S1-20. Fig. S1. Illustration of Nanopore systematic errors. Fig. S2. Illustration of homologs for polishing systematic errors. Fig. S3. Numbers of indels and mismatches of Bacillus. Fig. S4. Numbers of indels and mismatches of K pneumonia. Fig. S5. Examples of feature vectors at insertion and deletion loci. Fig. S6. Illustration of systematic errors in homopolymers. Fig. S7. Comparison of min-max normalization with one-hot encoding. Fig. S8. Illustration of minor strain variations at multiple loci. Fig. S9. Comparison of feature vectors at deletion and insertion loci. Fig. S10. Comparison of genome completeness of seven bacteria. Fig. S11. Comparison of genome completeness of the R9.4 Zymo dataset. Fig. S12. Whole-genome comparison of the P vulgaris CCU063. Fig. S13. Whole-genome comparison of the P vulgaris GOKU. Fig. S14. Correlation of genome quality with respect to the number of related genomes. Fig. S15. Whole-genome comparison of the P aeruginosa. Fig. S16. Whole-genome comparison of the S enterica. Fig. S17. Comparison of pseudogenes in Zymo R9.4 dataset. Fig. S18. Comparison of pseudogenes in Zymo R10.3 dataset. Fig. S19. Comparison of PEPPER in Zymo R10.3 dataset. Fig. S20. Evaluation of model accuracy. [file 13059_2021_2282_MOESM1_ESM.pdf]

Homopolish: a method for the removal of systematic errors in nanopore sequencing by homologous polishing (Supplementary Figures S1-20)

Yao-Ting Huang[1], Po-Yu Liu[2,3,4], and Pei-Wen Shih[1]

[1]*Department of Computer Science and Information Engineering, National Chung Cheng University, Chiayi, Taiwan.*
[2]*Department of Infectious Diseases, Taichung Veterans General Hospital, Taichung, Taiwan.*
[3]*Rong Hsing Research Center for Translational Medicine, National Chung Hsing University, Taichung, Taiwan.*
[4]*Ph.D. Program in Translational Medicine, National Chung Hsing University, Taichung, Taiwan.*

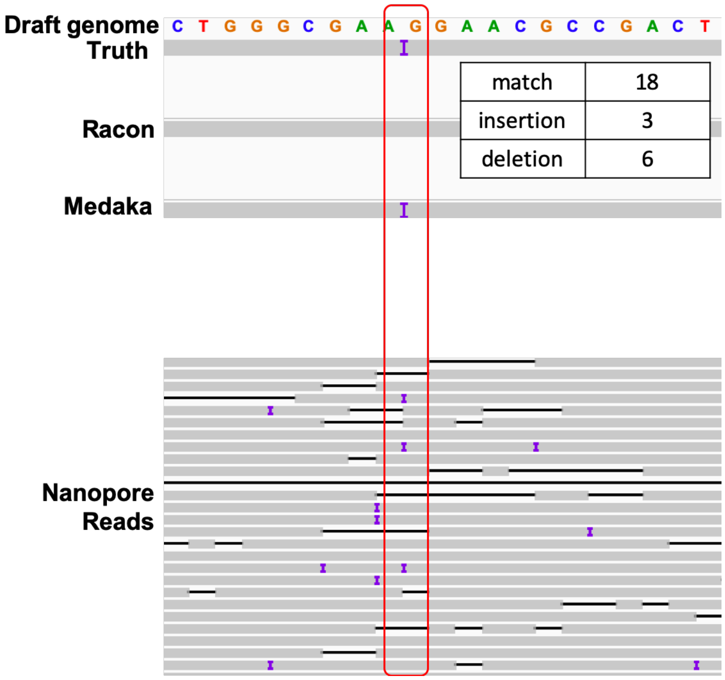

Fig. S1: Illustration of Nanopore systematic errors after polishing by Racon and by Medaka. The top shows the genomes before and after polishing. The bottom shows the IGV read alignments. Because majority of reads suggested no insertions at this locus (i.e., 18 vs 3 reads), Racon's majority-based strategy is unable to fix this error. Medaka successfully correct this systematic error by adding the missed nucleotide.

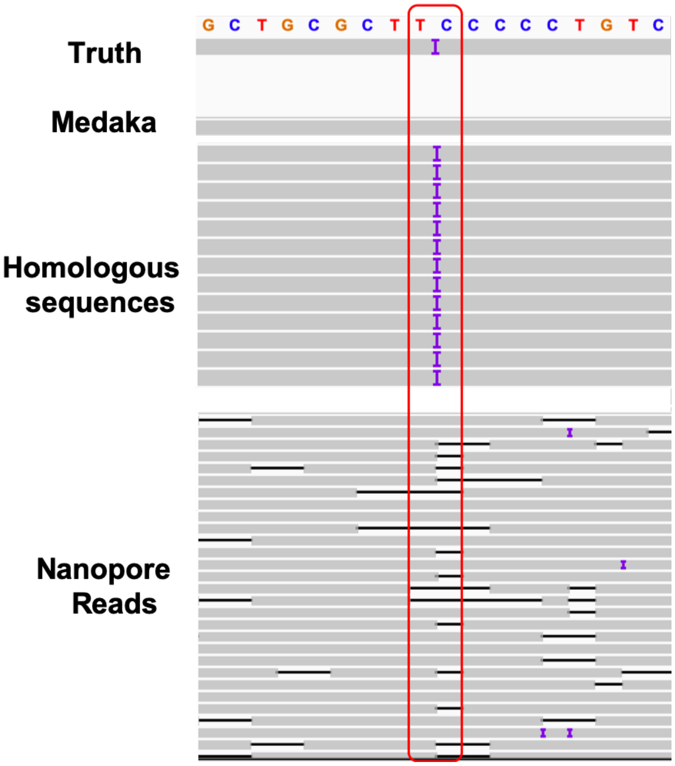

Fig. S2: Illustrations of homologous sequences for polishing Nanopore systematic errors. Because all the reads indicate no insertion at this locus (see bottom), Medaka failed to correct this sort of systematic errors. On the other hand, the homologous sequences at this region all agreed on an insertion at this locus, which in turn fix this systematic error.

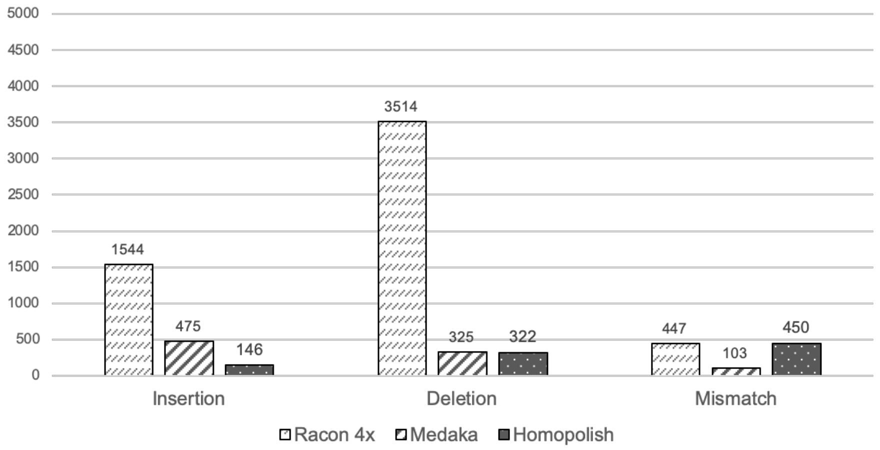

Fig. S3: Numbers of indels and mismatches of *Bacillus* genome after polishing by Racon, by Medaka, and by Homopolish.

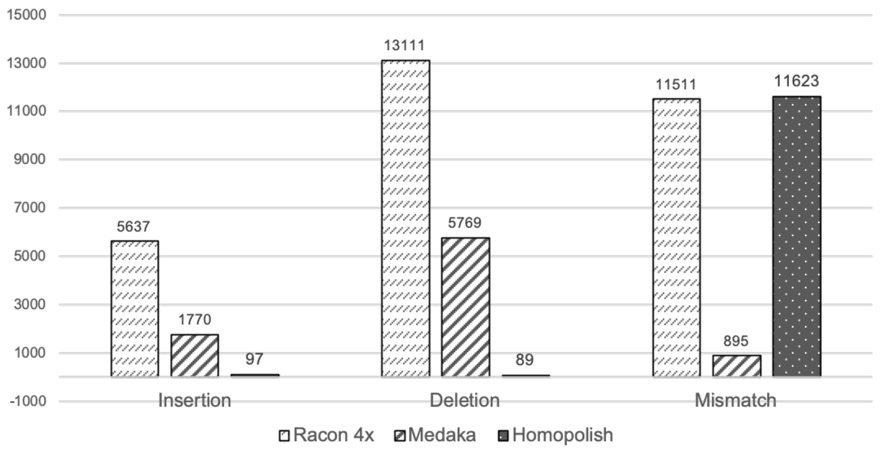

Fig. S4: Numbers of indels and mismatches of *K pneumonia* SAWA genome after polishing by Racon, by Medaka, and by Homopolish.

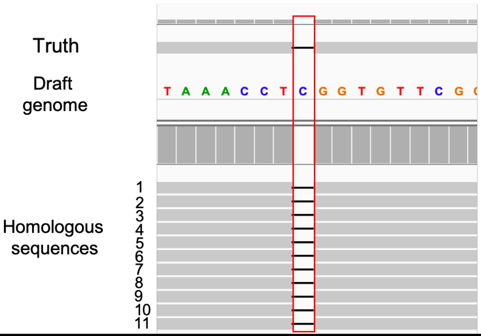

A	T	C	G	gap	Ins_A	Ins_T	Ins_C	Ins_G	Coverage	Label
0	0	0	0	11	0	0	0	0	11	Deletion

(a) Example of a deletion alignment profile and corresponding feature vector

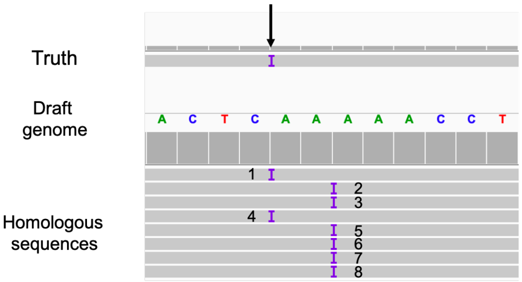

A	T	C	G	gap	Ins_A	Ins_T	Ins_C	Ins_G	Coverage	Label
0	0	0	0	0	2	0	0	0	8	Insertion A

(b) Example of an insertion alignment profile and corresponding feature vector

Fig. S5: Example of feature vectors for insertions and deletions. The allele counts of each feature are shown in the corresponding count tables.

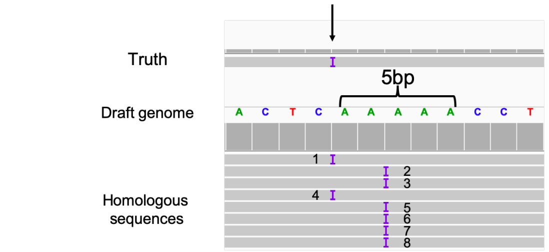

A	T	C	G	gap	Ins_A	Ins_T	Ins_C	Ins_G	Coverage	Homopolymer length
0	0	0	0	0	2	0	0	0	8	5

(a) A systematic error surrounding a homopolymer

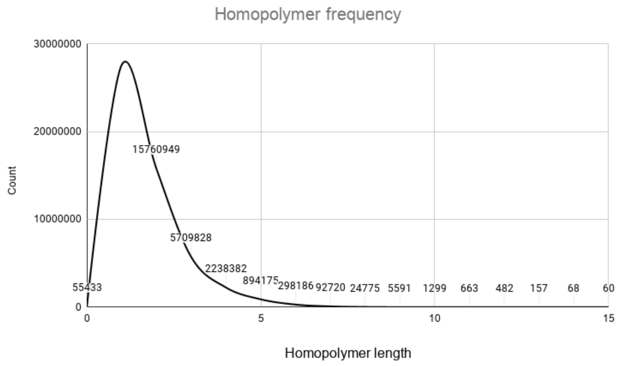

(b) Frequency distribution of homologous lengths

Fig. S6: Illustration of Nanopore systematic errors surrounding homopolymers. (a) An insertion error at the start of a homopolymer run of five adenine bases; (b) the frequency distribution of homopolymer lengths in the genome.

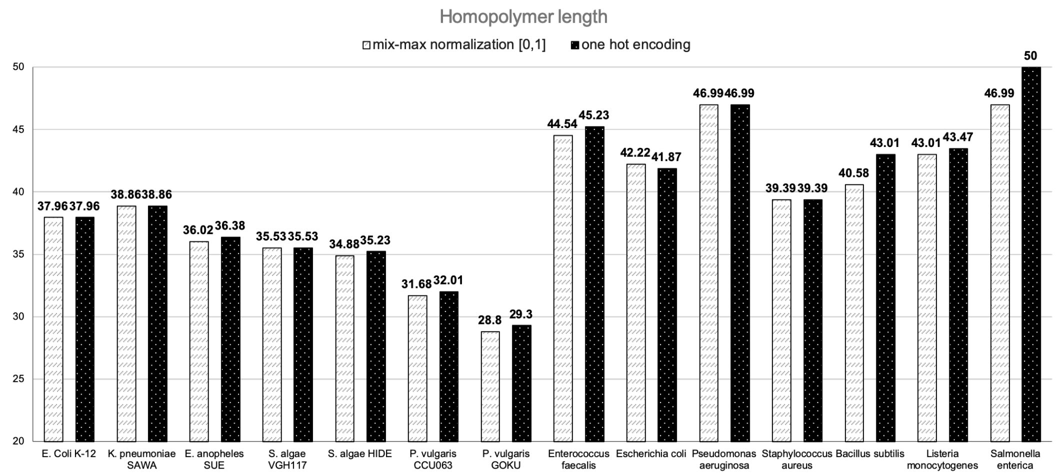

Fig. S7: Comparison of min-max normalization and one-hot encoding of the homopolymer length feature across fourteen bacteria. The accuracy is measured by the median Q scores.

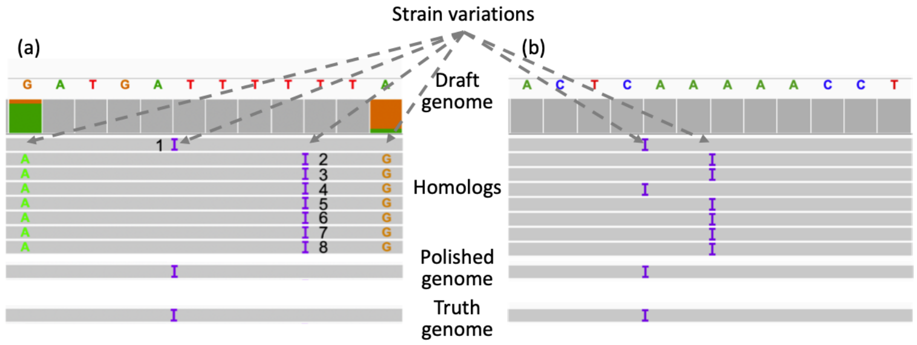

Fig. S8: Illustration of minor strain variations at multiple loci. A total of eight homologs were shown and majority of them suggest potential errors at these loci. (a) An example of distinct homologous similarity. The homologous sequence 1 flanking the minor allele is more similar to the draft genome, while those flanking the major allele contain mismatches and insertions. (b) An example of identical similarity. The homologous sequences flanking the major and the minor alleles both differ by one insertion when compared with the draft genome.

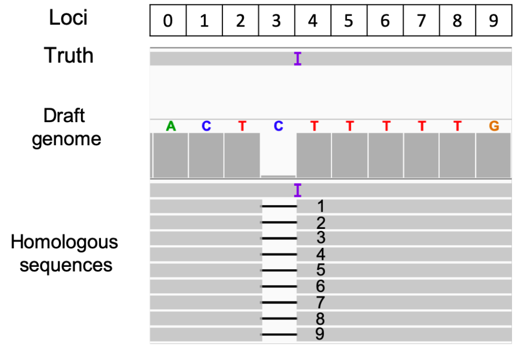

Feature Vectors

Loci	A	T	C	G	gap	Ins_A	Ins_T	Ins_C	Ins_G	Coverage	Label
3	0	0	0	0	9	0	0	0	0	10	No Correction
3	0	0	0	0	0	0	1	0	0	10	T

Fig. S9: Comparison of feature vectors of a deletion and an insertion loci. The feature vector containing the deletion alleles is distant from that containing the insertion allele.

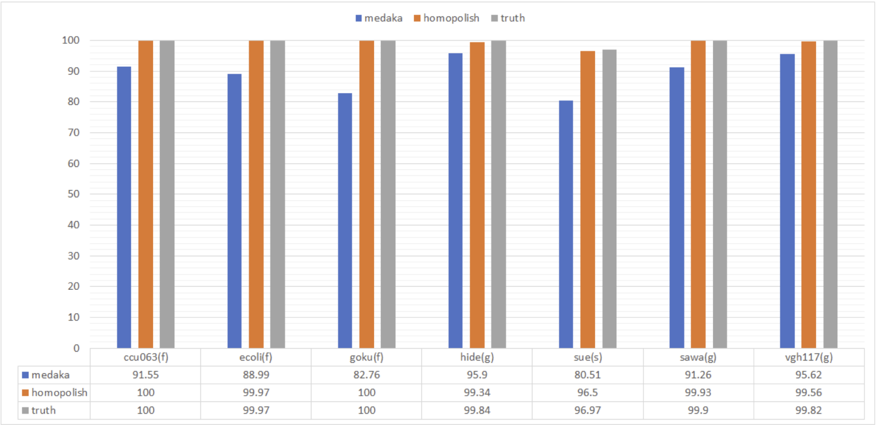

Fig. S10: Comparison of genome completeness (CheckM) of the seven bacteria isolates.

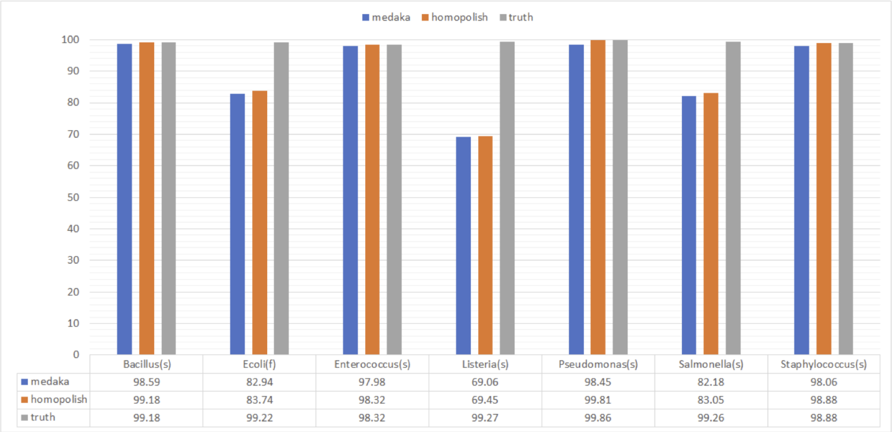

Fig. S11: Comparison of genome completeness (CheckM) of the R9.4 Zymo metagenomic dataset.

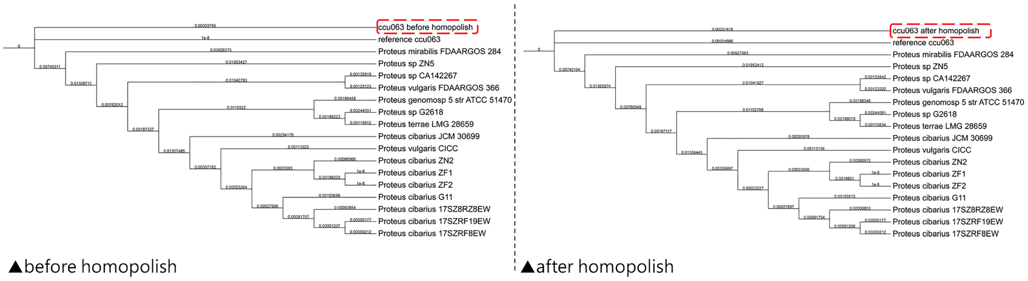

Fig. S12: Comparison of the whole-genome phylogeny of the *P vulgaris* CCU063, truth genome, and sixteen related genomes before and after Homopolish correction. The most closely-related genomes were retrieved from NCBI by Mash (>95% identity).

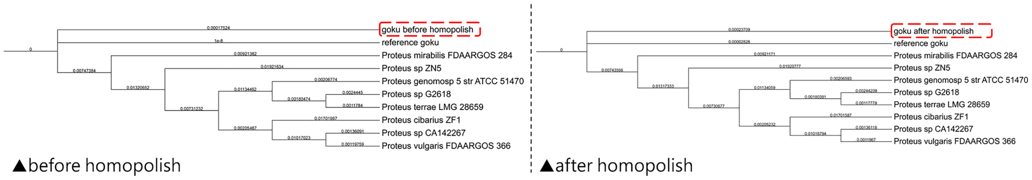

Fig. S13: Comparison of the whole-genome phylogeny of the *P vulgaris* GOKU, truth genome, and eight related genomes before and after Homopolish correction. The most closely-related genomes were retrieved from NCBI by Mash (>95% identity).

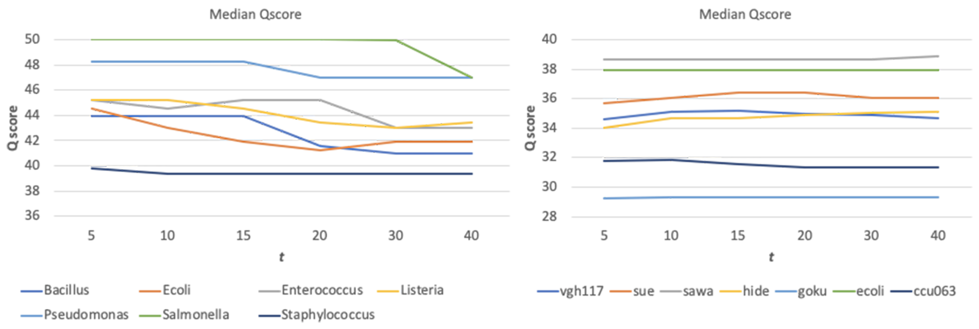

Fig. S14: The correlation of genome quality (median Q score) with respect to the maximum number of related genomes (t) retrieved by Homopolish.

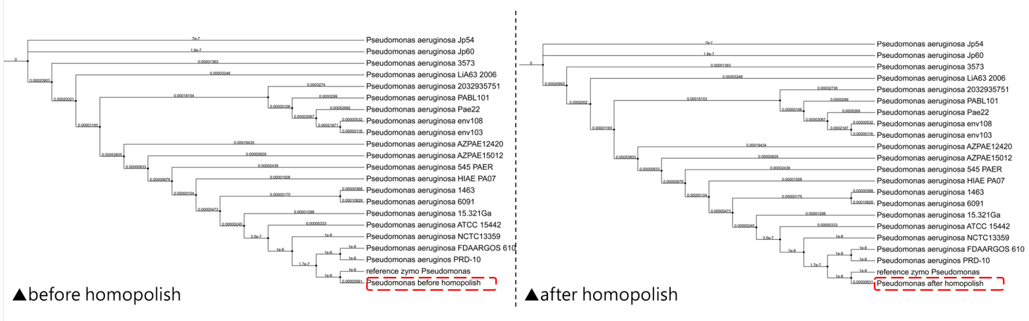

Fig. S15: Comparison of whole-genome phylogeny of *P aeruginosa*, its truth genome, and most closely-related genomes before and after running Homopolish. The top twenty similar genomes were retrieved from NCBI by Mash (>95% identity).

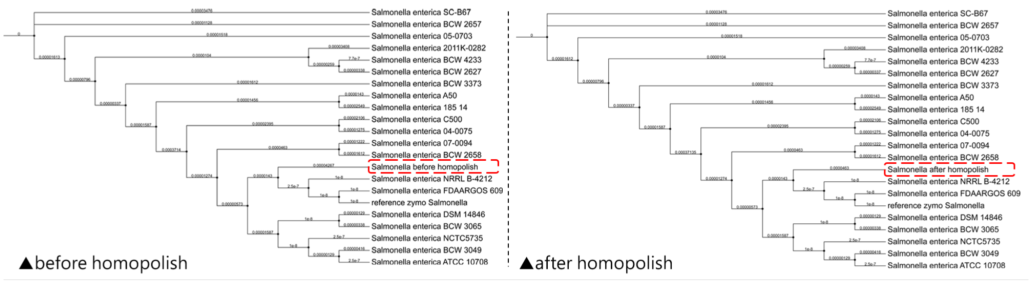

Fig. S16: Comparison of whole-genome phylogeny of *S enterica*, its truth genome, and most closely-related genomes before and after running Homopolish. The top twenty similar genomes were retrieved from NCBI by Mash (>95% identity).

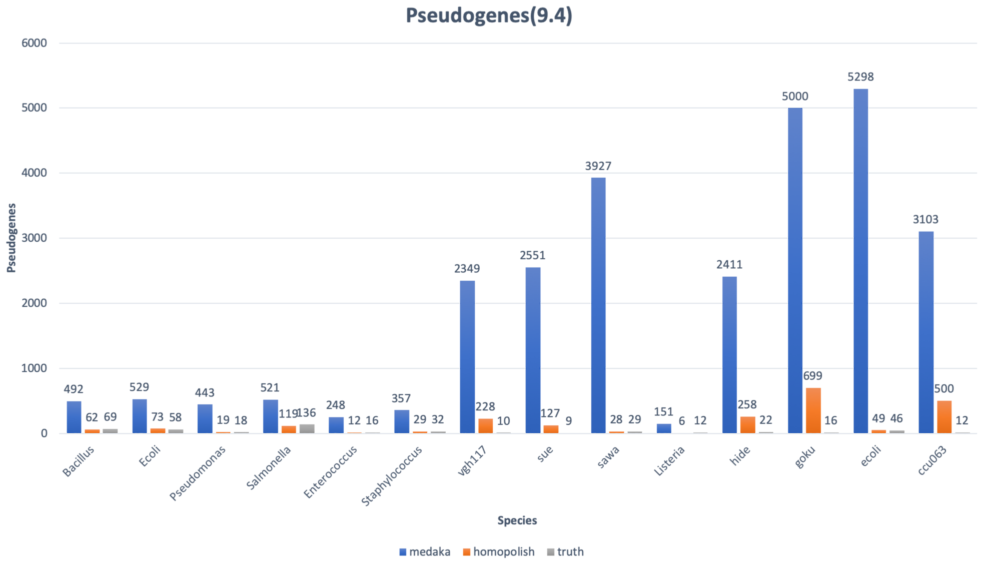

Fig. S17: Comparison of the numbers of pseudogenes in the genomes polished by Medaka, Homopolish, and the truth genomes in the Zymo metagenomic R9.4 dataset. The pseudogenes were annotated by DFAST.

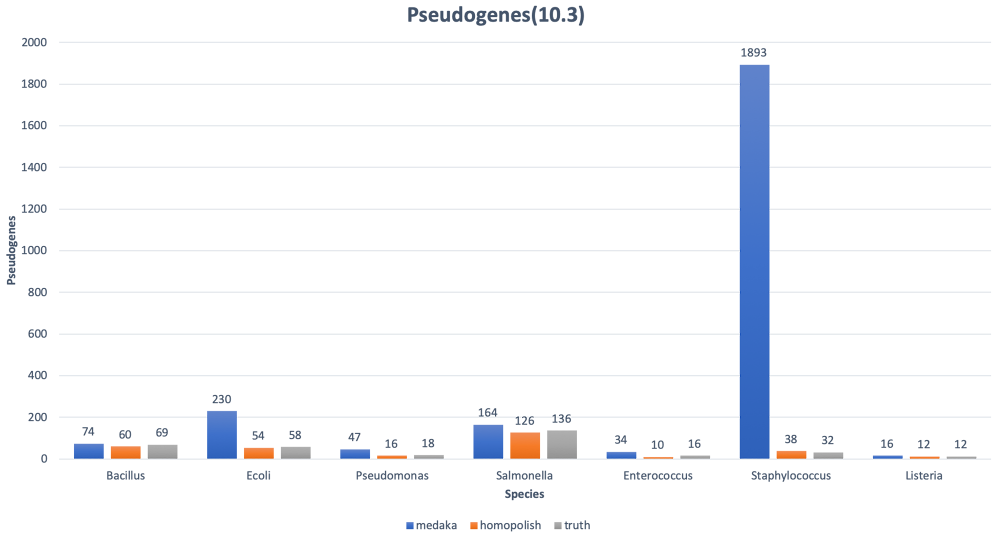

Fig. S18: Comparison of the numbers of pseudogenes in the genomes polished by Medaka, Homopolish, and the truth genomes in the Zymo metagenomic R10.3 dataset. The pseudogenes were annotated by DFAST.

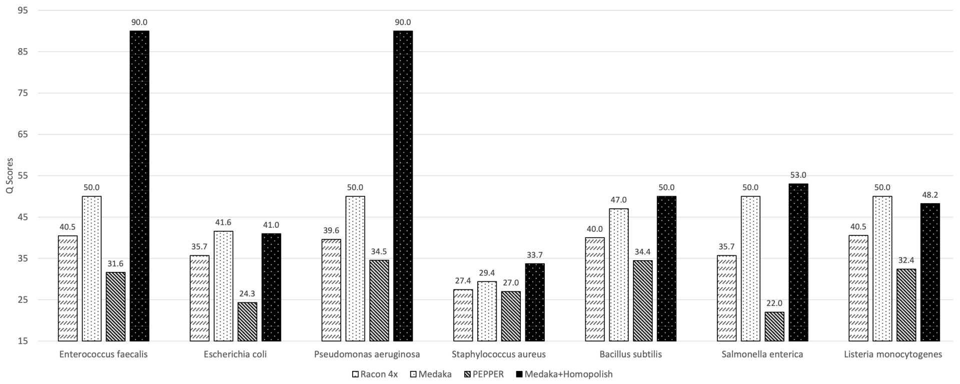

Fig. S19: Comparison of genome quality (Q score) polished by Racon, Medaka, PEPPER, and Homopolish on the R10.3 metagenomic dataset from Zymo Microbial Community Standard.

(a)

Label	Precision	Recall	F1-score
ins_A	0.94	0.97	0.96
ins_T	0.92	0.96	0.94
ins_C	0.87	0.97	0.91
ins_G	0.83	0.93	0.88
Deletion	0.90	0.93	0.92
No deletion	0.97	0.96	0.97
No insertion	0.95	0.87	0.91
Macro avg.	0.91	0.94	0.92

(b)

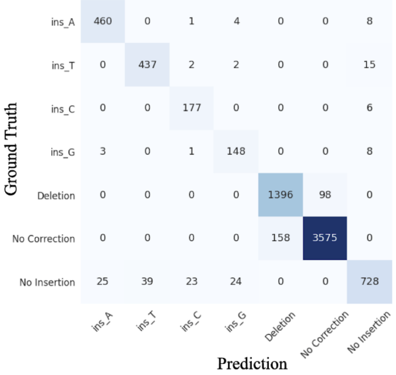

Fig. S20: (a) Macro average of precision, recall, and F1-score of prediction of seven classes. (b) Confusion matrix of testing set during model training.
