# Supplementary material for: Homopolish: a method for the removal of systematic errors in nanopore sequencing by homologous polishing
Source: Genome Biol. 2021 Mar 31;22:95. doi: 10.1186/s13059-021-02282-6 (PMC8011154; doi:10.1186/s13059-021-02282-6)
Supplement: Supplementary file 2 — Additional file 2 Additional file 2 includes Supplementary Tables S1-10. Table S1. Nanopore sequencing of six bacteria. Table S2. Illumina sequencing of six bacteria. Table S3. Nanopore sequencing of metagenomic datasets. Table S4. Hybrid assembly of six bacteria. Table S5. Metagenomic assembly of Zymo datasets. Table S6. Nanopore-only assembly of all isolates. Table S7. Comparison of polishing tools over the R9.4 Zymo dataset. Table S8. Comparison of polishing tools over the R9.4 bacterial dataset. Table S9. Comparison of polishing tools over the R10.3 Zymo dataset. Table S10. Comparison of label frequencies. [file 13059_2021_2282_MOESM2_ESM.pdf]

Homopolish: a method for the removal of systematic errors in nanopore sequencing by homologous polishing (Supplementary Tables S1-10)

Yao-Ting Huang[1], Po-Yu Liu[2,3,4], and Pei-Wen Shih[1]

[1]*Department of Computer Science and Information Engineering, National Chung Cheng University, Chiayi, Taiwan.*
[2]*Department of Infectious Diseases, Taichung Veterans General Hospital, Taichung, Taiwan.*
[3]*Rong Hsing Research Center for Translational Medicine, National Chung Hsing University, Taichung, Taiwan.*
[4]*Ph.D. Program in Translational Medicine, National Chung Hsing University, Taichung, Taiwan.*

Table S1: Nanopore sequencing statistics of six bacteria. The read numbers, N50 read length, maximum read length, and sum of total read bases are listed for each strain.

Nanopore sequencing				
Species	No. Reads	N50	Max	Sum
K. pneumoniae SAWA	406,263	17,529	122,092	842,252,640
E. anopheles SUE				
S. algae VGH117				
S. algae HIDE	178,071	25,496	141,550	2,944,017,556
P. vulgaris CCU063	161,898	10,271	49,577	1,292,396,754
P. vulgaris GOKU	33,172	9,292	79,283	163,089,117

Table S2: Illumina sequencing statistics of six bacteria. The read numbers, N50 read length, maximum read length, and sum of total read bases are listed for each strain.

Illumina sequencing				
Species	No. Reads	N50	Max	Sum
K. pneumoniae SAWA	2,204,942	151	151	332,946,242
E. anopheles SUE	2,518,328	151	151	380,267,528
S. algae VGH117	2,295,502	151	151	346,620,802
S. algae HIDE	2,166,486	151	151	327,139,386
P. vulgaris CCU063	2,647,252	151	151	399,735,052
P. vulgaris GOKU	2,723,350	151	151	411,225,850

Table S3: Nanopore sequencing statistics of two public metagenomic datasets (ZymoBIOMICS Microbial Community Standard) by Loman Lab (Nicholls et al., 2019). The read numbers, N50 read length, maximum read length, and sum of total read bases are listed for each dataset.

Version	No. Reads	N50	Max	Sum
R9.4	3,238,505	5,331	320,098	13.63Gb
R10.3	1,160,526	26,245	249,289	4.4Gb

Table S4: Statistic of reference genomes of in-house sequenced isolates (ground truth) reconstructed via hybrid assembly. The contig numbers and genome sizes are listed for each strain.

Isolate dataset ground truth		
Species	No. Contigs	Genome size
K. pneumoniae SAWA	1	5,403,374
E. anopheles SUE	1	4,201,198
S. algae VGH117	1	4,796,801
S. algae HIDE	1	4,950,784
P. vulgaris CCU063	1	4,141,308
P. vulgaris GOKU	1	4,141,320

Table S5: Statistics of metagenomic assembly from the ZymoBIOMICS dataset

Assembly data		
Species	No. contigs	Genome size
Enterococcus faecalis	1	2,867,861
Escherichia coli	1	4,780,323
Pseudomonas aeruginosa	1	6,800,605
Staphylococcus aureus	1	2,737,456
Bacillus subtilis	1	4,069,116
Salmonella enterica	1	4,774,301
Listeria monocytogenes	1	3,013,178

Table S6: Assembly statistics of public and in-house isolates by Nanopore-only sequencing.

Assembly data		
Species	No. contigs	Genome size
K. pneumoniae SAWA	1	5,419,444
E. anopheles SUE	1	4,214,907
S. algae VGH117	1	4,809,726
S. algae HIDE	1	4,963,570
P. vulgaris CCU063	1	4,153,753
P. vulgaris GOKU	1	4,172,433
E. coli K-12	1	4,637,729

Table S7: Comparison of Q scores and numbers of mismatches/indel errors for Flye, Racon, Medaka, MarginPolish, HELEN, and Homopolish over the ZymoBIOMICS Microbial Community Standard metagenomic dataset sequenced by R9.4 flowcells

Species	Methods	Q score	Median Qscore	Mismatchs	Insertions	Deletions
Enterococcus faecalis	Flye	22.1	22.07	389	17007	253
	Racon 4x	27.12	28.51	414	1303	3808
	Racon 4x + Medaka	37.18	37.7	131	253	157
	Racon 4x + Hompolish	31.74	38.24	420	57	1431
	MarginPolish	33.74	33.98	69	754	372
	MarginPolish + HELEN	41.87	43.98	34	63	86
	MarginPolish + Homopolish	43.47	50	71	33	23
	Racon 4x + Medaka + Homopolish	42.06	45.23	132	25	19
	MarginPolish + HELEN + Homopolish	43.67	50	53	27	41
Escherichia coli	Flye	25.23	25.46	603	11115	310
	Racon 4x	30.33	31	685	488	2531
	Racon 4x + Medaka	35.2	37.96	527	422	260
	Racon 4x + Hompolish	36.33	39.39	697	122	112
	MarginPolish	34.31	36.02	464	521	494
	MarginPolish + HELEN	35.63	38.54	477	229	384
	MarginPolish + Homopolish	37.48	41.55	484	132	97
	Racon 4x + Medaka + Homopolish	37.24	41.87	521	130	104
	MarginPolish + HELEN + Homopolish	37.44	41.25	471	127	120
Pseudomonas aeruginosa	Flye	26.39	26.5	738	14394	439
	Racon 4x	32.78	33.19	622	538	2417
	Racon 4x + Medaka	36.54	37.96	476	704	324
	Racon 4x + Hompolish	39.43	43.01	622	24	128
	MarginPolish	34.93	35.85	449	1096	637
	MarginPolish + HELEN	41.48	46.99	149	78	256
	MarginPolish + Homopolish	40.74	50	440	7	126
	Racon 4x + Medaka + Homopolish	40.55	46.99	474	4	120
	MarginPolish + HELEN + Homopolish	42.81	90	205	5	146
Staphylococcus aureus	Flye	22.88	22.9	876	12911	177
	Racon 4x	29.96	29.94	365	1012	1341
	Racon 4x + Medaka	35.79	36.2	247	274	190
	Racon 4x + Hompolish	37.31	37.83	367	38	96
	MarginPolish	33.07	33.37	232	845	253
	MarginPolish + HELEN	38.1	38.7	61	172	184
	MarginPolish + Homopolish	38.52	40	232	23	124
	Racon 4x + Medaka + Homopolish	38.69	39.39	247	24	93
	MarginPolish + HELEN + Homopolish	41.25	44.58	63	21	118
Bacillus subtilis	Flye	23.31	23.31	347	18254	347
	Racon 4x	28.66	28.64	447	1544	3514
	Racon 4x + Medaka	36.51	37.21	103	475	325
	Racon 4x + Hompolish	36.44	36.78	450	146	322
	MarginPolish	34.77	35.23	36	677	635
	MarginPolish + HELEN	37.28	37.7	30	317	409
	MarginPolish + Homopolish	40.6	45.23	49	102	201
	Racon 4x + Medaka + Homopolish	40.17	43.01	103	95	191
	MarginPolish + HELEN + Homopolish	40.83	43.98	31	89	214
Salmonella enterica	Flye	25.23	25.25	203	11446	280
	Racon 4x	30.72	31.08	339	448	2611
	Racon 4x + Medaka	37.3	38.86	190	340	217
	Racon 4x + Hompolish	40.18	43.01	340	14	31
	MarginPolish	34.81	35.53	63	637	623
	MarginPolish + HELEN	37.77	38.54	36	214	417
	MarginPolish + Homopolish	45.31	50	70	20	28
	Racon 4x + Medaka + Homopolish	42.51	50	186	13	26
	MarginPolish + HELEN + Homopolish	46.72	50	36	13	36
Listeria monocytogenes	Flye	22.65	22.58	174	11034	120
	Racon 4x	27.5	28.81	267	1277	2153
	Racon 4x + Medaka	31.82	38.54	94	669	605
	Racon 4x + Hompolish	31.87	38.54	266	540	545
	MarginPolish	31.24	36.2	42	846	675
	MarginPolish + HELEN	32.03	39.68	44	655	603
	MarginPolish + Homopolish	32.68	46.99	42	538	540
	Racon 4x + Medaka + Homopolish	32.48	43.47	94	538	541
	MarginPolish + HELEN + Homopolish	32.67	46.99	44	539	540

Table S8: Comparison of Q scores and numbers of mismatches/indel errors for Flye, Racon, Medaka, MarginPolish, HELEN, and Homopolish on bacterial isolates datasets sequenced by R9.4 flowcells

Species	Methods	Avg Q score	Median Q score	Mismatches	Insertions	Deletions
E. Coli K-12	Flye	22.09	22.25	14241	6248	8193
	Racon 4x	21.68	21.83	10743	3349	17450
	Racon 4x + Medaka	25.85	26.22	708	830	10537
	Racon 4x + Hompolish	25.92	26.31	10742	201	917
	MarginPolish	21.94	22.11	10742	2076	16893
	MarginPolish + HELEN	20.77	20.73	8918	4200	21383
	MarginPolish + Homopolish	25.92	26.35	10738	191	941
	Racon 4x + Medaka + Homopolish	34.15	37.96	708	181	894
	MarginPolish + HELEN + Homopolish	26.18	26.19	9210	290	716
K. pneumoniae SAWA	Flye	20.79	20.78	16431	22205	6255
	Racon 4x	22.49	22.49	11511	5637	13111
	Racon 4x + Medaka	28.06	28.1	895	1770	5769
	Racon 4x + Hompolish	26.6	26.52	11623	97	89
	MarginPolish	22.8	22.8	9778	3405	14925
	MarginPolish + HELEN	22.27	22.22	7904	4776	19117
	MarginPolish + Homopolish	27.31	27.33	9874	64	88
	Racon 4x + Medaka + Homopolish	37.34	38.86	896	57	43
	MarginPolish + HELEN + Homopolish	28.14	28.18	7983	74	232
E. anopheles SUE	Flye	22.25	22.2	1363	18718	5039
	Racon 4x	23.57	23.57	1163	6097	11215
	Racon 4x + Medaka	26.43	26.48	566	1114	7891
	Racon 4x + Hompolish	33.11	33.57	1224	384	443
	MarginPolish	25.57	25.61	389	1055	10198
	MarginPolish + HELEN	23.89	23.91	669	4614	11753
	MarginPolish + Homopolish	35.8	37.21	447	222	435
	Racon 4x + Medaka + Homopolish	35.32	36.38	611	216	407
	MarginPolish + HELEN + Homopolish	33.57	34.44	723	342	774
S. algae VGH117	Flye	23.74	23.7	1307	15982	3054
	Racon 4x	26.48	26.53	1262	3103	6423
	Racon 4x + Medaka	29.58	29.69	558	1028	3695
	Racon 4x + Hompolish	32.3	33.01	1399	562	863
	MarginPolish	28.08	28.11	582	989	5896
	MarginPolish + HELEN	27.6	27.66	523	1370	6449
	MarginPolish + Homopolish	33.76	34.69	702	407	907
	Racon 4x + Medaka + Homopolish	34.57	35.53	639	340	696
	MarginPolish + HELEN + Homopolish	33.59	35.02	659	424	1014
S. algae HIDE	Flye	23.97	23.92	1122	15674	2937
	Racon 4x	26.16	26.16	1034	2459	8414
	Racon 4x + Medaka	29.7	29.79	465	1013	3790
	Racon 4x + Hompolish	32.42	33.23	1189	508	1117
	MarginPolish	28.24	28.23	438	854	6088
	MarginPolish + HELEN	27.87	27.95	391	1187	6453
	MarginPolish + Homopolish	34.48	35.02	571	284	897
	Racon 4x + Medaka + Homopolish	35.05	35.23	541	283	714
	MarginPolish + HELEN + Homopolish	33.91	34.26	552	369	1076
P. vulgaris CCU063	Flye	23.5	23.56	783	14945	2819
	Racon 4x	24.21	24.2	1047	2767	11904
	Racon 4x + Medaka	27.68	27.75	345	458	6266
	Racon 4x + Hompolish	28.72	29.05	1716	1228	2620
	MarginPolish	26.58	26.62	170	874	8064
	MarginPolish + HELEN	26.58	26.68	169	1283	7663
	MarginPolish + Homopolish	31.18	31.46	635	662	1861
	Racon 4x + Medaka + Homopolish	31.51	32.01	710	567	1647
	MarginPolish + HELEN + Homopolish	30.71	31.02	711	767	2040
P. vulgaris GOKU	Flye	20.56	20.5	1501	33165	2059
	Racon 4x	20.91	20.85	3337	22954	7497
	Racon 4x + Medaka	23.97	24.02	1315	12180	3136
	Racon 4x + Hompolish	25.85	26.5	4087	4745	1939
	MarginPolish	22.49	22.53	1127	17146	5168
	MarginPolish + HELEN	26.58	26.68	169	1283	7663
	MarginPolish + Homopolish	27.77	28.39	1846	3685	1397
	Racon 4x + Medaka + Homopolish	28.77	29.3	1830	2572	1096
	MarginPolish + HELEN + Homopolish	24.32	24.84	4979	4526	4887

Table S9: Comparison of Q scores, mismatches, insertions, and deletions produced by Flye, Racon, Medaka, HELEN, and Homopolish on the metagenomic dataset and one isolate (E coli K12 MG1655) sequenced by R10.3 flowcells

Species	Methods	Avg. Q score	Median Q score	Mismatches	Insertions	Deletions
Enterococcus faecalis	Flye	39.45	40	70	59	194
	HELEN	45.02	90	58	25	5
	Racon 4x	39.52	40.46	94	116	108
	Racon 4x + Medaka	44.54	50	57	21	22
	Racon 4x + Medaka + Homopolish	45.2	90	57	23	6
Escherichia coli	Flye	34.45	35.45	749	175	659
	HELEN	37.7	41.25	564	83	155
	Racon 4x	34.46	35.69	920	237	533
	Racon 4x + Medaka	37.86	41.55	559	84	129
	Racon 4x + Medaka + Homopolish	38.15	40.97	547	92	83
Pseudomonas aeruginosa	Flye	36.57	37.96	480	149	864
	HELEN	40.85	53.01	411	12	132
	Racon 4x	37.5	39.59	591	104	511
	Racon 4x + Medaka	40.46	50	420	48	142
	Racon 4x + Medaka + Homopolish	41.01	90	420	0	118
Staphylococcus aureus	Flye	28.39	28.57	999	2750	195
	HELEN	35.24	36.58	86	95	633
	Racon 4x	27.43	27.43	1474	3020	428
	Racon 4x + Medaka	29.46	29.38	1112	1595	371
	Racon 4x + Medaka + Homopolish	33.42	33.77	1118	12	106
Bacillus subtilis	Flye	38.09	39.21	25	92	506
	HELEN	41.23	45.23	22	113	170
	Racon 4x	38.34	40	69	162	362
	Racon 4x + Medaka	41.68	46.99	20	86	169
	Racon 4x + Medaka + Homopolish	41.97	50	20	80	157
Salmonella enterica	Flye	34.24	34.44	456	623	708
	HELEN	40.54	42.22	44	93	283
	Racon 4x	35.73	35.69	380	403	489
	Racon 4x + Medaka	46.73	50	30	34	37
	Racon 4x + Medaka + Homopolish	48.32	53.01	30	23	17
Listeria monocytogenes	Flye	32.91	42.22	54	535	942
	HELEN	33.15	46.99	57	544	845
	Racon 4x	32.72	40.54	87	604	909
	Racon 4x + Medaka	33.32	50	45	527	820
	Racon 4x + Medaka + Homopolish	33.26	48.24	46	535	833
E coli K12 (isolate)	Racon 4x + Medaka	35.97	46.99	23	221	928
	Racon 4x + Medaka + Homopolish	36.28	90	23	182	887
	HELEN	40.54	42.22	44	93	283

Table S10: Comparison of label frequencies before and after removing duplicate feature vectors

	Frequency	
Label	before	after
Insertion A	4,683	4,683
Insertion T	4,463	4,463
Insertion C	1,799	1,799
Insertion G	1,625	1,625
Deletion	14,935	14,935
No Deletion	30,208,536	38,020
No Insertion	7,849	7,849
