# Supplementary material for: Homopolish: a method for the removal of systematic errors in nanopore sequencing by homologous polishing
Source: Genome Biol. 2021 Mar 31;22:95. doi: 10.1186/s13059-021-02282-6 (PMC8011154; doi:10.1186/s13059-021-02282-6)
Supplement: Supplementary file 3 — Additional file 3 Additional file 3 includes the review history. [file 13059_2021_2282_MOESM3_ESM.docx]

Review History

**First round of review**

**Reviewer 1**

**Were you able to assess all statistics in the manuscript, including the appropriateness of statistical tests used?** Yes

**Were you able to directly test the methods?** Yes

**Comments to author:**

In the presented manuscript authors present a novel method Homopolish for polishing of the ONT assemblies with homologous sequences.

The method is based on the SVM prediction approach with features extracted from comparison of targeted assembly and the homologous assemblies.

Authors demonstrate the proposed method's utility on several bacterial, viral and fungi genomic datasets, and show evidence of Homopolish outperforming state-of-the art polishing tools, such as Medaka and HELEN.

Authors also demonstrate various method-combination variations, allowing the reader to clearly understand and appreciate the benefit of the ensemble/pipeline type approach for ONTassebmly polishing.

Authors do a good job in presenting and walking through the comparative results which demonstrate that Homopolish if not alone, but in combination with other polishing methods, may become a go-to method for ONT assembly polishing.

The methodological part of the paper is lacking in both clarity and rigor.

The main concerns come from determination of the "true" assemblies (which were obtained by a hybrid Unicycler assembler strategy) but no detailed analysis of potential systemic errors in said true assemblies is reported, and its effects on the quality assessments downstream are thus unclear for the reader.

Furthermore, it is unclear from the text whether or not the Homopolish training dataset and comparison/verification datasets are identical/overlap, or distinct, which in case of identical/overlap raises a question of applicability outside of the considered organisms, as questions of overfitting, method's applicability range, etc arise.

Also, no real analysis is done on the phylogenetic (or similar) distance between the targetted assembly and the related genomes used for polishing.

Similar concern can be raised in terms of the number "t" of similar genomes used for polishing, which has a default of 20, as a a reader can benefit from a clearer demonstration of the dependency between both evolutionary/phylogenetic relationships between genomes considered for polishing.

While results with default parameters seem to perform very well, understanding the limitations related to parameters' selection strategies either on reported organisms, or on the assemblies of other genome's classes, is missing.

Overall, the area of ONT assembly polishing with no need for additional sequencing experiments (i.e., hybrid sequencing/assembly approach) is of great interest to the community, but the breadth and depth of analysis/reporting of the proposed method's applicability can be improved to clarify the presented results, as well as attract a bigger audience and make the method more generally adopted.

Also, the manuscript would benefit from proofreading native language speaker, as to improve readability (some, but not all, examples are provided in the minor comments below). Here one of the main problems is mixing of times, which sometimes make it harder to distinguish parts of the text that either relate to the method's development or to the discussed comparison/performance results, or both.

Minor:

------

* abstract: effective --> effectively

* background: ultra-long reads of ONT has demonstrated its power --> have demonstrated their power

* background: increase in accuracy that the previous version --> ... over the previous version

* background: We observed homologous sequences from closely-related... --> this sentence requires rephrasing to clarify its meaning and fix language

* results: ... by homopolish, we want to know --> rephrase

* results: we show that Homopolsih can improve the quality of early-generation genomes --> this sentence requires a rewrite with more specifiers (tech, etc) put into it

* results: when comparison with HELEN --> when comparing with HELEN

* exclusion of HELEN results for R10.3 is somewhat justifiable, but if not too much work, adding it would benefit the reader; though this is not a stopper for me

* conclusion: this paper developed --> rephrase

* methods: a number of features were extracted from the alignments --> a rephrase of this sentence would benefit the reader

* methods: which can be parallelly polished in a multicore environment --> which can be polished in a multithreaded environment in parallel

* figures are missing Y-size labels

* labels on bars in figures often overlap neighboring labels/bars. rotation of the bar labels my improve clarity of the figures.

**Reviewer 2**

**Were you able to assess all statistics in the manuscript, including the appropriateness of statistical tests used?** yes

**Were you able to directly test the methods?** No

**Comments to author:**

I read with interest the article entitled "High-Quality Genomes of nanopore Sequencing by Homologous Polishing". The authors have developed a method that they have dedicated to the polishing of draft assemblies. Their method is based on comparative genomics and as such is truly original. They compared their software with existing tools, such as Medaka and HELEN, on a large number of bacterial genomes and metagenomic samples. Even though the underlying algorithm is really interesting, I think the application is not suitable.

First, the article has a lot of typos (see a short-list below), the title is unclear and is a list of keywords, the figures are too simple and not easy to visualize, leading to the impression that the article was written too quickly.

More importantly, the proposed polishing method is only based on the draft genome and did not take into account the sequencing reads. This is an important characteristic which is only mentioned on page 4. By using comparative genomics, Homopolish will tend to decrease the variability between genome assemblies. As an example, if a genome contains a true deletion, which corresponds to an inactivated gene, Homopolish will restore the coding frame observed in close genomes. Even though the method makes sense, as the error rate is still high today, it can lead to unwanted results. Moreover this approach is only valid in coding regions (or at least regions under selective pressure), and the authors have not discussed the polishing effect in non coding regions, and they shyly discuss the problem of SNPs. Indeed their method could not correct mismatches, as SNPs are common and often not share across close genomes..

I suggest that the authors use Homopolish to detect potential assembly errors, in bacterial and eukaryotic genomes and especially in coding regions, rather than polish draft assemblies.

A short list of typos:

-P2L13 : "NarginPolish"

P3L22 : Please provide an URL for fastmer

P3L48 : "mismatched"

P3L48 "Homopolsh"

P4L22 "The is because"

P4L38: "Raon"

P4L39 "K penumoiae"

P4L41 "When comparison…"

P6L10 "polishing polishing models"

Legend Table S3 : "R10.4 flowcells"

**Authors Response**

**Point-to-Point Responses**

*Major Comments of Reviewer #1:*

1. *In the presented manuscript authors present a novel method Homopolish for polishing of the ONT assemblies with homologous sequences. The method is based on the SVM prediction approach with features extracted from comparison of targeted assembly and the homologous assemblies. Authors demonstrate the proposed method's utility on several bacterial, viral and fungi genomic datasets, and show evidence of Homopolish outperforming state-of-the-art polishing tools, such as Medaka and HELEN. Authors also demonstrate various method-combination variations, allowing the reader to clearly understand and appreciate the benefit of the ensemble/pipeline type approach for ONT assembly polishing. Authors do a good job in presenting and walking through the comparative results which demonstrate that Homopolish if not alone, but in combination with other polishing methods, may become a go-to method for ONT assembly polishing.*

**Ans:** We thank the reviewer’s encouragement.

2. *The methodological part of the paper is lacking in both clarity and rigor. The main concerns come from determination of the "true" assemblies (which were obtained by a hybrid Unicycler assembler strategy) but no detailed analysis of potential systemic errors in said true assemblies is reported, and its effects on the quality assessments downstream are thus unclear for the reader.*

**Ans:** We are sorry that the original sentences are misleading. The majority of “true” genomes were retrieved directly from NCBI without hybrid assembly (i.e., the two Zymo metagenomic datasets, virus, fungi, and R9.4/10.3 *E coli* ). Only the true genomes of six additional strains (sequenced by ourselves) were constructed by the hybrid assembly. Because the reference genomes of these strains are not available, hybrid assembly is the only way to build the

“not-really-true” genomes. Thus, the accuracy of this hybrid dataset (Q30-40) is lower than other datasets using true NCBI genomes (Q40-50).

This revision provides a reference-free assessment, genome completeness (by CheckM), over this hybrid dataset (Supplementary Figure S10, also shown below). Briefly, a set of marker genes (e.g., housekeeping genes) were selected by CheckM for assessing the genome quality of each tool. HELEN was not included due to far worse accuracy. The completeness of Homopolish (~97-100%) is not only higher than that of Medaka (~81-95%) but also quite close to that of truth genomes (~97-100%). We also investigated the R9.4 metagenomic dataset with ground truth genomes available (Supplementary Figure S11). Homopolish again improves the completeness over Medaka. However, the completeness of several strains is unreasonably low (e.g., ~69% in *Listeria* ) due to MetaFlye misassembly (confirmed by Canu assembly, data not shown). Consequently, we would like to note that the CheckM completeness is still not a perfect assessment.

Supplementary Figure S10. Comparison of genome completeness (CheckM) of the seven bacteria isolates.

Supplementary Figure S11. Comparison of genome completeness (CheckM) of the R9.4 Zymo metagenomic dataset.

Inspired by Reviewer 2’s comments, we found the number of pseudogenes can be a reference-free assessment as well, because the (uncorrected) indel errors often lead to frameshifts, producing an excessive amount of pseudogenes. We computed the number of pseudogenes in fourteen genomes (polished by Medaka/Homopolish) via DFAST (Supplementary Figure S17). The results indicated pseudogenes in Homopolished genomes are quite similar to those of the truth genomes (e.g., 62 vs 69 in *Bacillus* ), while those polished by Medaka all have excessive amounts of pseudogenes (e.g., 492 in *Bacillus* ). Therefore, the analysis of pseudogene amounts also confirmed that the genome quality of Homopolish is not only higher than that of Medaka but also close to that of truth genomes.

Supplementary Figure S17. Comparison of the numbers of pseudogenes in the genomes polished by Medaka, Homopolish, and the truth genomes of 14 bacteria in the metagenomic/isolate (R9.4) dataset. The pseudogenes were annotated by DFAST.

It is our view that the existing reference-free assessments (e.g., CheckM/BUSCO/ideel/pseudogene), though less affected by misassemblies/variations, are not sensitive enough to reflect the true accuracy of each program. For instance, we have observed many genomes can obtain ~100% completeness even with only ~Q30 accuracy. On the other hand, reference-based assessments (e.g., Q scores of fastmer), though affected by hybrid errors, can measure the accuracy of each method at a higher resolution. Thus, the reference-based assessment was chosen as the main metric throughout the paper. We hope the reviewer could see Homopolish can further improve the genome quality in terms of both reference-based and reference-free assessments.

3.	*Furthermore, it is unclear from the text whether or not the Homopolish training dataset*

*and comparison/verification datasets are identical/overlap, or distinct, which in case of identical/overlap raises a question of applicability outside of the considered organisms, as questions of overfitting, method's applicability range, etc arise.*

Ans: We apologize for missing these important details. The training set of the R9.4 model (basecalled by Guppy 2.2) includes the following seven strains: *P aeruginosa* , *E coli* , *S*

*aureus* , and *E faecalis, K pneumoniae* , *P vulgaris* VGH117, *P vulgaris* GOKU, whereas the other seven bacterial strains, one fungus, and one virus are validation datasets. The training set of the R10.3 model includes four species: *P aeruginosa, E coli, S aureus, E. faecalis,* whereas the remaining four species are validation set. Overall, the accuracy of Homopolish outperforms Medaka and HELEN regardless of training or validating datasets (Figures 2 and 4, attached below with training and validation labelling). The training and validation details have been added into the revised manuscript (“Model training” in the Method section) .

Figure 2. Comparison of genome quality (Q score) polished by Racon, Medaka, Homopolish (R), MarginPolish, HELEN, and Homopolish (M) on the R9.4 metagenomic dataset. Medaka and Homopolish (R) are run after Racon. HELEN and Homopolish (M) are invoked after MarginPolish.

Figure 4. Comparison of genome quality (Q score) polished by Racon, Medaka, Homopolish (R), MarginPolish, HELEN, and Homopolish (M) on seven bacterial isolates. Medaka and Homopolish (R) are run after Racon. HELEN and Homopolish (M) are invoked after MarginPolish.

Besides, we think our model is not (or at least less) overfitting in comparison with others. First, even without training on distant fungi and viruses, Homopolish still outperforms the others (Table 3 in the main paper). Second, Medaka and HELEN trained many models dependent on multiple parameters (e.g., MinIon or PromethIon, HAC or fast basecalling,

Guppy version, R9.4 or R10.3, ...). On the contrary, we only trained two models with one parameter (R9.4 and R10.3), regardless of the basecaller or platforms. Finally, w e investigated the applicability of our method on recent basecaller (Guppy 3.5 on R9.4 flow cell) using a strain (*E coli* K12 DH10B) kindly provided by our collaborator. Note that our model was trained on data basecalled by Guppy 2.2 or earlier version. The results (see below) indicates that Homopolish improves the genome from Q45 to Q90. The excessive number of insertion errors is due to a large (~1.3kb) insertion sequence not on the reference (i.e., strain variation, data not shown). We hope these could answer the reviewer's concern of overfitting.

|  | Avg Q score | Median Q score | Insertions | Deletions |
| --- | --- | --- | --- | --- |
| **Flye** | 29 | 31.61 | 1586 | 3638 |
| **Racon** | 32.81 | 36.68 | 1563 | 702 |
| **Medaka** | 34.65 | 45.23 | 1450 | 72 |
| **Homopolish** | 34.96 | 90 | 1357 | 37 |

4. *Also, no real analysis is done on the phylogenetic (or similar) distance between the targetted assembly and the related genomes used for polishing. Similar concern can be raised in terms of the number "t" of similar genomes used for polishing, which has a default of 20, as a reader can benefit from a clearer demonstration of the dependency between both evolutionary/phylogenetic relationships between genomes considered for polishing. While results with default parameters seem to perform very well, understanding the limitations related to parameters' selection strategies either on reported organisms, or on the assemblies of other genome's classes, is missing.*

**Ans:** We thank the reviewer for the constructive suggestions. The isolate dataset also aimed to investigate our program on polishing common (e.g., *K pneumoniae* ), less common (e.g., *S algae* ), and rare (e.g., *P vulgaris* ) species (Figure 5). We reconstructed the whole-genome phylogenies of two rare species (*P vulgaris* CCU063 and GOKU) along with their related genomes (Supplementary Figures S12 and S13, attached below). *P vulgaris* GOKU and CCU063 only fetched eight and sixteen related genomes (>95% Mash identity) from NCBI, respectively. Although Homopolish still improved the quality of these two genomes (Q27 to Q32 and Q24 to Q29), the degree of improvement is lower than polishing other common species (e.g., Q26 to 38 in *K pneumoniae* ). In Figure 5 (attached below), the genome quality decreases from common (Q38), less common (Q35), to rare species (Q29).

Supplementary Figure S12. Whole-genome phylogeny of the *P vulgaris* CCU063, truth genome, and sixteen related genomes retrieved from NCBI by Mash (>95% identity).

Supplementary Figure S13. Whole-genome phylogeny of the *P vulgaris* GOKU, truth genome, and eight related genomes retrieved from NCBI by Mash (>95% identity).

The parameter *t* (maximum number of retrieved genomes) has been tested over a range (5~40). Supplementary Figure S14 (attached below) plots the Q scores w.r.t *t* in fourteen bacteria. Although this parameter can be further optimized for particular species (e.g., *t*=5 is highest in *Pseudomonas* ), it looks to us there is not much difference. When *t*>20, the quality of most species deteriorates. The default value ( *t*=20) was chosen as it balances well for both common or rare species.

Supplementary Figure S14. The correlation of genome quality (median Q score) with the maximum number of related genomes ( *t*) retrieved by Homopolish.

In summary, the efficacy of Homopolish correction is correlated with the abundance of related genomes in NCBI (i.e., common or rare). The improvement of polishing common species is larger than rare species (Figure 5, attached below). Moreover, Homopolish is less

sensitive to the parameter *t* as it adaptively corrects errors or retains variations according to local conservation (i.e., selection pressure). A new section discussing the limitations of our method (e.g., non-coding regions, plasmids) has been included in the revised manuscript (i.e., “Limitations of homologous polishing” in Discussion). We hope this could help the reviewer understand the strengths and weaknesses of our method.

Figure 5. Comparison of genome quality (Q score) polished by Medaka, Medaka+Homopolish, HELEN, and HELEN+Homopolish on the isolate dataset. Although Homopolish can improve Medaka or HELEN, the degree of improvement is decreased from common (e.g., *E coli*) to rare species (e.g., *P vulgaris* ).

5.	*Overall, the area of ONT assembly polishing with no need for additional sequencing*

*experiments (i.e., hybrid sequencing/assembly approach) is of great interest to the community, but the breadth and depth of analysis/reporting of the proposed method's applicability can be improved to clarify the presented results, as well as attract a bigger audience and make the method more generally adopted. Also, the manuscript would benefit from proofreading native language speaker, as to improve readability (some, but not all, examples are provided in the minor comments below). Here one of the main problems is mixing of times, which sometimes make it harder to distinguish parts of the text that either relate to the method's development or to the discussed comparison/performance results, or both.*

**Ans:** We thank the reviewer again for many valuable suggestions. More analysis has been added in the revision to show the strengths and weaknesses of Homopolish ( “Limitations of homologous polishing” and “Preservation of strain variations” in Discussion, Supplementary Figures S10-20). In particular, as suggested in the reviewer’s minor comments, HELEN and PEPPER (next version of HELEN, under development) on the R10.3 data have been included in the revised manuscript (Figure 6 and Supplementary Figure S19, shown below). Figures 2-6 were refined accordingly (i.e., adding labels and increasing spacing). The entire manuscript has been proofread by a language editing service.

Figure 6. Comparison of genome quality (Q score) polished by Racon, Medaka, HELEN, and Homopolish on the R10.3 metagenomic dataset from Zymo Microbial Community Standard. HELEN was run after MarginPolish, Medaka was launched after Racon, and Homopolish was invoked after Medaka.

Supplementary Figure S19. Comparison of genome quality (Q score) polished by Racon, Medaka, PEPPER, and Homopolish on the R10.3 metagenomic dataset from Zymo Microbial Community Standard.

*Comments of Reviewer #2:*

*I read with interest the article entitled "High-Quality Genomes of nanopore Sequencing by Homologous Polishing". The authors have developed a method that they have dedicated to the polishing of draft assemblies. Their method is based on comparative genomics and as such is truly original. They compared their software with existing tools, such as Medaka and HELEN, on a large number of bacterial genomes and metagenomic samples. Even though the underlying algorithm is really interesting, I think the application is not suitable.*

Ans: We thank the reviewer’s interests in this work. Below please find the point-to-point responses to the reviewer’s comments.

1. *First, the article has a lot of typos (see a short-list below), the title is unclear and is a list
   of keywords, the figures are too simple and not easy to visualize, leading to the impression that the article was written too quickly.*

**Ans:** We are sorry for those typos. The manuscript has been proofread by a language editing service. The title has been changed to “Removal of systematic errors in Nanopore sequencing by homologous polishing.” Figure 1 is replaced with the illustration of our method highlighting the two most important features: removal of errors and retention of variations. Many new figures have been included in the revision for showing the strengths and weaknesses of our method (Supplementary Figures S10-20).

Figure 1. Illustration of Homopolish workflow. Homopolish retrieves closely-related genomes by screening MinHash sketches of NCBI RefSeq microbial genomes. Homologs within these genomes were extracted by genome alignments. An SVM was trained for the removal of errors and retention of variations by mining features from the homologous pileup.

2. *More importantly, the proposed polishing method is only based on the draft genome and
   did not take into account the sequencing reads. This is an important characteristic which is only mentioned on page 4. By using comparative genomics, Homopolish will tend to decrease the variability between genome assemblies. As an example, if a genome contains a true deletion, which corresponds to an inactivated gene, Homopolish will*

*restore the coding frame observed in close genomes. Even though the method makes sense, as the error rate is still high today, it can lead to unwanted results.*

**Ans:** We are sorry that the reviewer’s concerns have been considered during development but not explained clearly in the previous manuscript. The model was designed and trained for distinguishing between sequencing errors and strain variations. Only errors will be removed while variations will be retained. Our favorite examples are shown below (also in Supplementary Figure S8). Although most homologs suggest potential errors at these loci, the truth genome reveals that they are strain variations with minor alleles. Homopolish is capable of retaining these minor variations via feature engineering (e.g., homopolymers, homologous similarity). The naive majority vote using homologs will result in even worse accuracy than Medaka (data not shown). A machine learning approach (trained for retaining strain variations) is necessary to make this idea work.

Supplementary Figure S8. Illustration of strain variations at multiple loci. A total of eight homologs were shown and the majority of them suggest potential errors at these loci. Homopolish was trained for retaining these minor variations without false corrections.

To prove that the strain variations were not decreased, we compared the whole-genome phylogenies before and after Homopolish correction using common and rare species. The twenty most closely-related genomes (>99% Mash identity) of two common species (*P aeruginosa* and *S enterica* in the Zymo dataset) were retrieved for whole-genome phylogeny reconstruction (see Method). Although their genetic distance is very close, the phylogenies remain the same before and after Homopolish (Supplementary Figures S15 and S16, see below). Similarly, the phylogenies of two rare species (*P vulgaris* CCU063 and GOKU) and their closely related genomes are also unchanged after Homopolish (see Supplementary Figures S12 and S13). Therefore, the phylogenetic distance between the polished and their related genomes are well preserved.

Supplementary Figure S15. Comparison of whole-genome phylogeny of *P aeruginosa* , its truth genome, and most closely-related genomes before and after running Homopolish. The top twenty similar genomes were retrieved from NCBI by Mash.

Supplementary Figure S16. Comparison of whole-genome phylogeny of *S enterica* , its truth genome, and twenty most closely-related genomes before and after running Homopolish. The top twenty similar genomes were retrieved from NCBI by Mash.

Besides, our method can preserve pseudogenes (i.e., inactivated genes) containing true indels. We compared the pseudogenes (annotated by DFAST) in the genomes polished by Medaka, Homopolish, and the truth genome using Loman’s Zymo R9.4/R10.3 datasets (Supplementary Figures S17 and S18, attached below). The Racon-Medaka pipeline produces excessive amounts of pseudogenes (e.g., 151-5298 in R9.4) due to quite a few uncorrected errors. On the contrary, the genomes polished by Homopolish yield not only much fewer pseudogenes (e.g., 10-126 in R9.4) but the numbers are also quite similar to those of truth genomes (e.g., 12-136 in R9.4). Further investigation reveals that the minor discrepancies between Homopolished genomes and the truth genome (e.g., 62 vs 69 in Bacillus) are due to pseudogenes that have been missed before Homopolish (possibly by Flye misassembly). Consequently, most pseudogenes are successfully preserved by our method.

Supplementary Figure S17. Comparison of the numbers of pseudogenes in the genomes polished by Medaka, Homopolish, and the truth genomes of 14 bacteria in the metagenomic/isolate (R9.4) dataset. The pseudogenes were annotated by DFAST.

Supplementary Figure S18. Comparison of the numbers of pseudogenes in the genomes polished by Medaka, Homopolish, and the truth genomes in the Zymo metagenomic R10.3 dataset. The pseudogenes were annotated by DFAST.

We hope the reviewer could see the strain variations, pseudogenes, and phylogenies are well preserved by the machine-learning approach. Without variation retention, the false corrections will lead to even worse genome quality. The revision has been rephrased to emphasize more on the variation retention (e.g., Figure 1, Supplementary Figures S8, S12-13, S15-18, “Preservation of strain variations” in Discussion). We thank the reviewer for the constructive comments.

3. *Moreover this approach is only valid in coding regions (or at least regions under selective pressure), and the authors have not discussed the polishing effect in non coding regions, and they shyly discuss the problem of SNPs. Indeed their method could not correct mismatches, as SNPs are common and often not share across close genomes. I suggest that the authors use Homopolish to detect potential assembly errors, in bacterial and eukaryotic genomes and especially in coding regions, rather than polish draft assemblies.*

Ans: We thank the reviewer’s suggestions. We started by polishing coding regions in the beginning. Later we observed that the extent of selection pressure is, though dynamic, larger than expected in non-coding regions. Table 4 compares the numbers of errors in coding and non-coding regions before and after running Homopolish. Although the correction ratios in coding regions are higher as expected (70-95%), the errors in non-coding regions can be still significantly reduced (28-97%). The large variance of correction ratios in non-coding regions is possibly due to species-specific selection pressures. We note again that the model only corrects the errors under strong pressure and retain variations under weak or no pressure. Therefore, we are confident it can be safely applied to the entire genome. Just the correction efficiency may be reduced in non-coding regions free of selection pressure.

Table 4: Comparison of numbers of indel errors in coding and non-coding regions before and after running Homopolish (over Zymo R9.4 dataset from Loman Lab ). DFAST was used for annotation of coding/non-coding regions in each genome, and errors in each region were counted by bedtools intersection.

|  | **Coding regions errors** | | | **Non-coding regions errors** | | |
| --- | --- | --- | --- | --- | --- | --- |
|  | **Before** | **After** | **Correction  ratio** | **Before** | **After** | **Correction  ratio** |
| ***B subtilis*** | 446 | 80 | 82.06% | 232 | 165 | 28.88% |
| ***E faecalis*** | 244 | 21 | 91.39% | 176 | 34 | 80.68% |
| ***S aureus*** | 306 | 38 | 87.58% | 100 | 17 | 83.00% |
| ***L monocytogenes*** | 160 | 19 | 88.13% | 80 | 26 | 67.50% |
| ***P aeruginosa*** | 666 | 30 | 95.50% | 279 | 8 | 97.13% |
| ***S enterica*** | 469 | 66 | 85.93% | 118 | 13 | 88.98% |
| ***E coli*** | 544 | 163 | 70.04% | 201 | 141 | 29.85% |

We agree with the reviewer that most SNPs are not under selection pressure and indistinguishable from errors, except for a few cases within extremely conserved regions (e.g., ribosomal genes). In practice, indel errors (instead of SNP errors) are the major obstacle to our projects, because existing annotation tools (e.g., Prokka/DFAST/PGAP) often miss

functionally-important genes due to indel-induced frameshifts. Therefore, the removal of indel errors is the main focus of this paper.

However, we respectfully disagree that it can only be used for the detection of assembly errors. It’s our view that, unless Nanopore can reach the accuracy of PacBio HiFi, there is always room for improvement by Homopolish. For example (Supplementary Table S9), the number of strain variations is close to or larger than that of errors in the R10.3 dataset with higher quality. For instance, only 48 insertion errors in *P* aeruginosa are left by Medaka. The results indicate that Homopolish can gain further improvement over Medaka (e.g., 48 to 0 insertions, 142 to 118 deletions). If our model was unable to distinguish between variations and errors, the genome quality would become much worse owing to false corrections of variations.

Supplementary Table S9 (an excerpt of *P aeruginosa* ). Comparison of Q scores, mismatches, insertions, and deletions produced by Flye, HELEN, Racon, Medaka, and Homopolish on the R10.3 dataset. Medaka was run after Racon, and Homopolish was invoked after Medaka.

|  | **Median Q score** | **Mismatches** | **Insertions** | **Deletions** |
| --- | --- | --- | --- | --- |
| **Flye** | 37.96 | 480 | 149 | 864 |
| **HELEN** | 53.01 | 411 | 12 | 132 |
| **Racon** | 39.59 | 591 | 104 | 511 |
| **Medaka** | 50 | 420 | 48 | 142 |
| **Homopolish** | 90 | 420 | **0** | **118** |

As a consequence, we hope the reviewer could see that homologs within conserved regions can accurately distinguish Nanopore systematic errors from strain variations by machine learning techniques. We thank the reviewer’s constructive suggestions of phylogeny, pseudogenes, and non-coding analysis, which improve the breadth and depth of this study. These results have been added into this revision (Table 4, Supplementary Figures S10-20, Two subsections, “Limitations of homologous polishing” and “Preservation of strain variations” in Discussion).

**Second round of review**

**Reviewer 1**

In the presented revision authors have addressed my questions and concerns and the updated version of the manuscript is improved, both in the depth of the presented analysis, as well as the structure/style of the information presentation.

I would like to highlight added method's applicability analysis, additional data/parameter application/investigation, as well as improved metrics.

Overall, the improved version of the manuscript is suitable for publication with minor style/grammar correction which can be done at the editorial level.

**Reviewer 2**

I have read the revised manuscript "Removal of Systematic Errors in Nanopore Sequencing by Homologous Polishing". I would like to congratulate the authors because the manuscript has been considerably improved both in form and in substance. My comments have been addressed and the authors modified the manuscript accordingly. I still have a few minor points that need to be checked before publication.

1. I have not found any evidence that the Nanopore and Illumina datasets produced by the authors are available through the NCBI or EBI databases..

2. The title is better, but I suggest replacing "Nanopore Sequencing" with "Nanopore Assemblies", which will make it clearer that Homopolish is not a long-read corrector.

3. Page2, lines 46-47, I suggest adding that training of a basecaller for a specific species is not an option for metagenomic sequencing data.

4. I would like the authors to point out that Homopolish has not been tested on non-compact genomes, in which non-coding regions represent an important part of the genome. These genomes, in fact a large proportion of eukaryotic genomes, could not be corrected to the same level as the small and compact genomes. Please add a sentence on page 6 (line 37) and perhaps a hint in the title.

**Authors Response**

**Point-by-point responses to the reviewers’ comments:**

I have read the revised manuscript "Removal of Systematic Errors in Nanopore Sequencing by Homologous Polishing". I would like to congratulate the authors because the manuscript has been considerably improved both in form and in substance. My comments have been addressed and the authors modified the manuscript accordingly. I still have a few minor points that need to be checked before publication.

1. I have not found any evidence that the Nanopore and Illumina datasets produced by the authors are available through the NCBI or EBI databases.

*Ans: The NCBI accession numbers are listed in the Availability of Data and Materials”*

*section, references [29-34], and the data are also uploaded to Github [35].*

2. The title is better, but I suggest replacing "Nanopore Sequencing" with "Nanopore Assemblies", which will make it clearer that Homopolish is not a long-read corrector.

*Ans: We decided to use the title suggested by editor as the source of systematic errors*

*are Nanopore sequencing instead of genome assemblies.*

3. Page2, lines 46-47, I suggest adding that training of a basecaller for a specific species is not an option for metagenomic sequencing data.

*Ans: Done*

4. I would like the authors to point out that Homopolish has not been tested on non-compact genomes, in which non-coding regions represent an important part of the genome. These genomes, in fact a large proportion of eukaryotic genomes, could not be corrected to the same level as the small and compact genomes. Please add a sentence on page 6 (line 37) and perhaps a hint in the title.

*Ans: Done*
